# Oedema as a prognostic factor for seizures in meningioma - a systematic review and meta-analysis

**DOI:** 10.1007/s10143-025-03416-1

**Published:** 2025-02-19

**Authors:** Matthew J. Tanti, Sarah Nevitt, Molly Yeo, William Bolton, Paul Chumas, Ryan Mathew, Melissa J. Maguire

**Affiliations:** 1 Present Address: Faculty of Medicine and Health, University of Leeds, Leeds, LS2 9JT UK; 2 Department of Neurology, Leeds Teaching Hospitals NHS Foundation Trust, Leeds, LS1 3EX UK; 3 Centre for Reviews and Dissemination, University of York, York, YO10 5DD UK; 4 Department of Neurosurgery, Leeds Teaching Hospitals NHS Foundation Trust, Leeds, LS1 3EX UK

**Keywords:** Meningioma, Epilepsy, Oedema, Prognostic factor, Surgery, Meta-analysis

## Abstract

Meningiomas are benign intracranial tumours that commonly lead to seizures and oedema. An understanding of seizure risk factors is essential for the meningioma community. Many studies have differing conclusions on whether oedema is associated with seizure. Existing meta-analyses are limited by lack of focus on oedema. Our objective was to summarise all literature on oedema as a prognostic factor for seizures in meningioma patients. We searched OVID, Scopus, Pubmed, Web of Science, ClinicalTrials.gov and Google scholar up to April 2024 for reports with more than 10 human meningioma participants. Statistics were performed on R-Studio. Cochrane and Campbell guides for systematic reviews and meta-analysis were followed. Risk of bias was assessed with ROBINS-E. Our protocol was uploaded to INPLASY. We included 51 studies for meta-analysis and 21 for narrative review. Most studies were of surgically treated adults. Heterogeneity was low once outliers were removed. Preoperative oedema was associated with preoperative seizure (*k* = 28, *n* = 7,725, *OR* 3.5, 95% *CI* = 3.1-4.0, *I2* = 0%, *p* < .001), early postoperative seizure (*k* = 9, *n* = 2,929, *OR* 1.5, *CI* = 1.1–1.9, *I2* = 0%, *p* = .011) and late postoperative seizure (*k* = 9, *n* = 2,150, *OR* 1.9, *CI* = 1.5–2.2, *I2* = 0%, *p* < .001). We performed an additional adjusted analysis for preoperative seizures which was also significant (*k* = 3, *n* = 2,241, *OR* 3.9, *CI* = 2.4–6.3, *I*2 = 0%, *p* = .007). There were few studies of post-radiosurgery oedema and seizure, and of postoperative oedema and seizure, with insignificant but positive associations. Preoperative oedema is a key factor for preoperative seizures. Oedema also increases risk of postoperative seizures. Further study in conservative, radiosurgery and paediatric populations, as well as study of oedema and seizure severity or subtype is warranted.

**Supplementary Information:**

The online version contains supplementary material available at 10.1007/s10143-025-03416-1.

## Introduction

Oedema and seizures are commonly seen in meningioma despite their extra-axial location and usual slow growth [1–3]. Seizures impair health and quality of life; a full understanding of risk factors will guide the meningioma community [4–9]. There are many studies of seizure risk factors in meningioma, but there are gaps in the literature [2]. Not all studies agree that oedema is a risk factor, particularly for postoperative seizures [2, 10–12]. Furthermore, there is no summary of oedema and seizure in conservative, radiosurgery or paediatric populations. Prior meta-analyses did not focus on oedema so advanced meta-analysis techniques such as subgrouping, regression, or adjusted analyses were not performed [2, 12]. Subgrouping or meta-regression can determine whether there are differences in strength of association by study level characteristics, such as geographical location of study or imaging modality. Adjusted analysis can determine whether oedema is significant despite other risk factors, such as absence of headache for preoperative seizures.

## Materials and methods

A full protocol was uploaded to INPLASY [13].

### Objectives

Our primary objective was to systematically review and meta-analyse oedema as a prognostic factor for seizures in all treatment populations. As secondary objectives, and by focusing on oedema, we explored the role of other study level characteristics in modifying this relationship. We also described other non-oedema factors in narrative and “covariate review” format.

### Study inclusion/exclusion

We used a PICOTS framework when reviewing reports for inclusion and exclusion (Table 1) [14]. Epilepsy and seizures are often used interchangeably, but ‘epilepsy’ should refer to a tendency for recurrent unprovoked seizures [15]. We included seizures whether described as seizure or epilepsy. Reports were included irrespective of study design, language, or peer review status.

**Table 1:** PICOTS summary for inclusion or exclusion

Population	Index prognostic factor	Comparator	Outcome	Timing	Setting
Human meningioma participants of any age Radiological or tissue diagnosis of meningioma Studies with less than 10 participants were excluded	Oedema before or after any treatment Measured by magnetic resonance imaging (MRI) or computed topography (CT) Can be defined as: • binary (absent versus present or threshold) • ordinal • continuous (e.g. volume) • oedema index (e.g. oedema volume divided by tumour volume)	Comparators were only considered for the adjusted meta-analysis^a^ We highlight potential comparators with narrative and covariate review	Isolated or recurrent seizures Can be defined as^b^: • binary (absent versus present or threshold) • ordinal • continuous	Oedema and/or seizure could have occurred: • pre-treatment • early post treatment (within a week) • late post treatment (after one week) Reports were excluded from the meta-analysis if they were unclear whether seizures occurred pre or post-treatment	Any management setting was considered: • conservative • surgery • radiosurgery

^a^Use of adjusted meta-analysis is additional to our INPLASY protocol

^b^All studies reported seizures as a binary outcome

### Study measures

A separate meta-analysis was performed for each time-point relative to treatment (surgery or radiotherapy):

- pre-treatment oedema and pre-treatment seizure
- pre-treatment oedema and post treatment seizure (early or late)
- post-treatment oedema and seizure

One week is currently used to distinguish early and late post-treatment seizures in the meningioma literature [16].

### Search methods

**Fig. 1:**
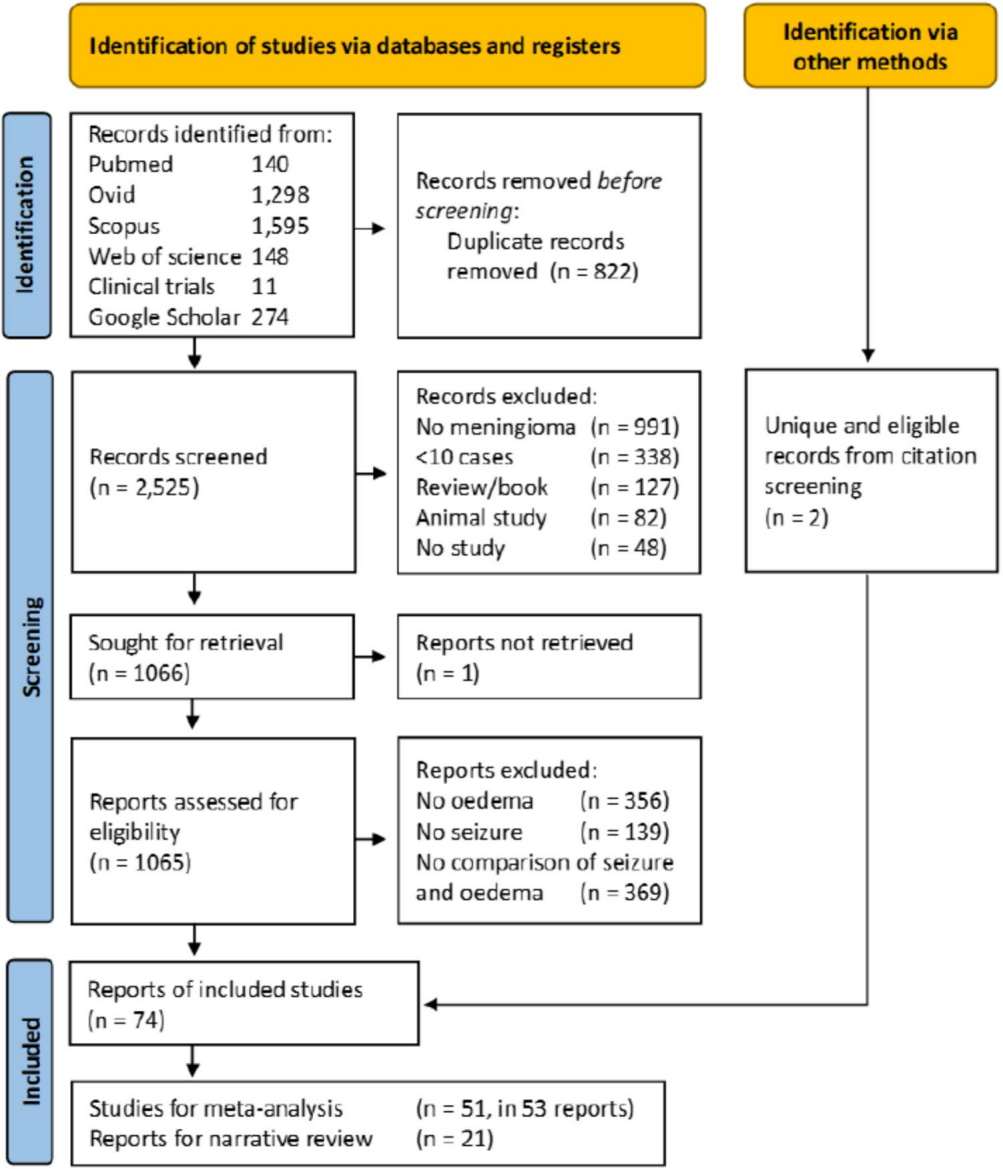
PRISMA flow diagram for study selection

An unfiltered search without date limitation was performed in April 2024 (updated since INPLASY protocol) using five databases in addition to Google scholar (Fig. 1) [13]. Search terms were optimised for each database (Online Resource 1).

### Data collection and analysis

Study selection phases:

1. One author screened titles and abstracts for exclusion (e.g. study of non-human participants). A random 10% sample was checked by a second author and no errors were found (updated method since INPLASY protocol) [13].
2. Full reports were then assessed for eligibility by two authors.

#### Data extraction

Two authors independently extracted outcomes for the meta-analysis. Most studies provided effect sizes (i.e. odds ratios [*OR*] and 95% confidence intervals [*CI*s]) or contingency tables as measures of association and defined oedema or seizures as being present or absent. Two studies grouped seizure status by average oedema area or volume; we estimated a standardised mean difference (*SMD*) and standard deviation (*SD*) and converted to *OR* and 95% *CI* using Campbell online calculators [17]. Data in figures was extracted with WebPlotDigitizer [18]. Unless stated otherwise we used unadjusted effect sizes due to factor selection variability in multivariable models across studies. One author extracted additional study details, for example number of patients, age, gender, and the methods used to measure oedema. Other factors associated with seizures in univariable and multivariable analyses were extracted from studies when both statistically significant and non-significant findings were reported. They are presented in our ‘Covariate Review.’ Two authors determined whether eligible studies could be included in the meta-analysis and or covariate review. If neither, they were included in the narrative review. Studies in the meta-analysis were also screened for additional seizure outcomes (e.g. seizure frequency, severity or semiology) and summarised in narrative format. When reports provided insufficient data for unadjusted meta-analysis, authors were contacted for further data.

#### Risk of bias assessment

Risk of bias was assessed by one author for all studies in the meta-analysis. The exposure outcome of observational studies form (ROBINS-E) and ROBVIS visualisation tool was used to create risk of bias figures [19, 20].

#### Statistical analysis

The R-project programming tool (R) version 4.1.2 was used for statistical analyses and figures (packages in Online Resource 2) [21].

##### Pooling effect sizes

In the meta-analyses, effect sizes were converted to the natural log of *OR* (ln*OR)* and its standard error (*SE*[ln*OR*]*)* [22, 23]. Studies with zero cells on contingency tables had a continuity correction of 0.5 added to all cells. We used a random-effects model with Hartung Knapp adjustment. The generic inverse variance method was used instead of the Mantel-Haenszel as raw binary data was not available for all studies (update to INPLASY) [13].

##### Heterogeneity

Between study heterogeneity was assessed with Higgins & Thompson’s *I2* statistic (< 25%: low heterogeneity, < 50%: moderate, < 75%: substantial) and the heterogeneity variance τ2 was assessed with the Paule-Mandel estimator [24]. The *SD* of true effect sizes (τ), Cochran’s Q and the *H2* statistic were also reported [24]. Prediction intervals were used to estimate future effect directions.

Subgroup meta-analysis and meta-regression were performed when more than 10 studies were present. We were able to review: risk of bias, infratentorial tumours, seizure definition, oedema modality, oedema definition and continent (latter for subgroup analysis only). For preoperative oedema and postoperative seizure, we used the same variables in addition to preoperative seizure, timing of postoperative seizure and use of prophylactic anti-seizure medication (ASM). Subgrouping and meta-regression are limited by examination of study level data; many factors of interest were not stratified by oedema and seizure status. Subgroup analyses, like the main meta-analyses, were based on complete cases. Complete case and multiple imputation were used for both univariable and multivariable meta-regression.

##### Sensitivity analysis and publication bias

Studies with 95% *CI* not overlapping the pooled *CI* were classed as outliers. Assessments of publication bias included, when possible, contour enhanced funnel plots, Egger’s tests, and corrections using the trim and fill and (without outliers) *p*-curve analyses.

### Summary of findings

We summarised our findings using the Grading of Recommendations, Assessment, Development, and Evaluations (GRADE) framework (addition to INPLASY) [25].

## Results

### Baseline characteristics

**Fig. 2:**
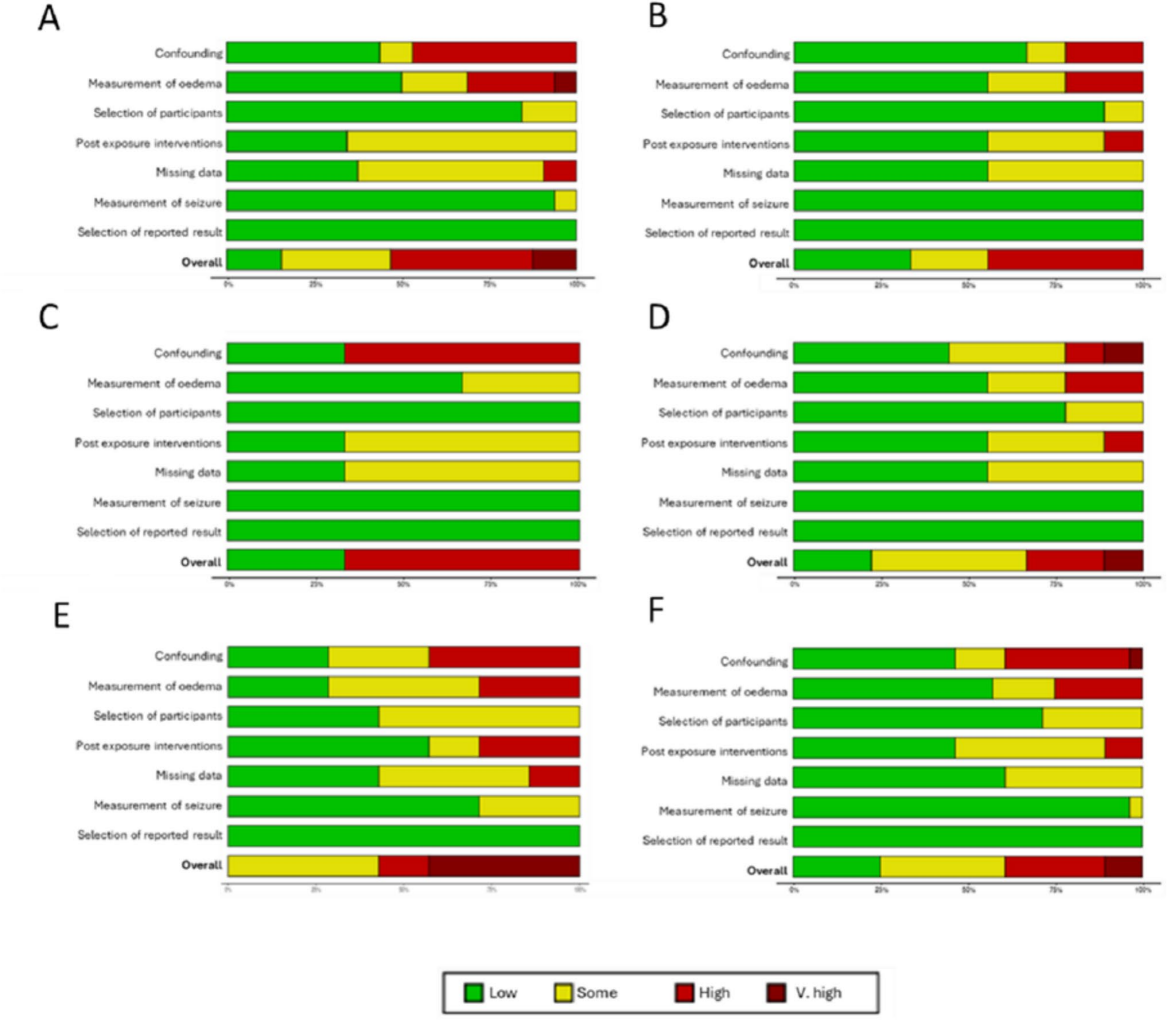
Risk of bias assessments: **A** Preoperative oedema and seizure; **B** Preoperative oedema and early postoperative seizure; **C** Post-radiosurgery oedema and seizure; **D **Preoperative oedema and late postoperative seizure; **E** Seizure and postoperative oedema; **F** Preoperative oedema and any postoperative seizure

In 74 reports were 53 studies (*k*) eligible for meta-analysis and 21 for narrative/covariate review (Fig. 1; Table 2) [26–99]. Overlapping populations were seen in 14 reports but most described different outcomes and only two were excluded from the meta-analysis [41, 69]. All studies were observational and apart from two all were retrospective [68, 85]. Reports originated mainly from European (*k* = 35), Asian (*k* = 22) or North American (*k* = 12) continents. Countries of origin included Germany (*k* = 14), United States of America (*k* = 12) and China (*k* = 8). Most studies (89%) were of surgical cohorts and the remainder had radiosurgery. Any grade of meningioma was included in most (75%). Inclusion years ranged from 1968 to 2023; most (58%) recruited within the previous 10 years. Most patients were female in the 6th or 7th decade of life. Preoperative oedema was seen in 49% of patients (*k* = 40, total *n* = 10,124). Oedema was identified by magnetic resonance imaging (MRI) in 72% of studies with a binary (55%) or threshold (32%) definition. Prophylactic ASM use was specified in 55% of reports; of which 49% of studies used them (ranging from 9 to 100% of patients), 41% did not, and the remainder (10%) had preoperative seizures (Online Resource 3). In studies that provided seizure proportions, 23% of patients had preoperative seizure (*k* = 30, total *n* = 7,785), 6% had early postoperative seizure (*k* = 8, *n* = 2,873), and 17% had late postoperative seizures (*k* = 9, *n* = 2,150). A description of seizure semiology, outcome, or definition was provided in 27% of reports (Online Resource 3). Pre-operative focal seizures were identified in 27–65% of patients with seizure (impaired awareness in 2–14%) and 36–51% had new postoperative focal seizures. Generalised seizures were noted in 34–68% preoperatively and new generalised seizures in 32–55% postoperatively. Most studies report long-term postoperative seizure freedom (Engel I or ILAE classification I) in approximately 80–90% of patients, decreasing to 70–80% in those with preoperative seizures. Many studies had a high risk of bias due to confounding factors or measurement of oedema (Fig. 2).

**Table 2:** Details of included studies

Author	Country	Inclusion period	Study overlap	Treatment	Total (*n*)	Females (*n*)	Age*	Paed-iatric	Who grade	Infra-tentorial	Imaging for oedema	Oedema definition	Threshold definition	Seizure inclusion criteria	Proph-ylactic ASM used?	Seizure definitions or description	Seizure follow up months (for late postop or post SRS)	Meta-analysis	Covariate review	Narrative only
Abzalova et al. 2023 [26]	Russia	2017–2020		Resection	56	47	63			None	MRI	Volume		No preop	Yes	Yes		Eps, POS	Eps	
Ahmed et al. 2023 [27]	USA			Resection	165	108	57		1 to 3	Yes	MRI	Binary								Pre
Ahmeti et al. 2023 [38]	Germany	2003–2019		Resection	696	526	60		1 to 3	Yes	MRI	Binary						Pre, Aps	Pre	
Asemota et al. 2022 [49]	USA	2010–2014		Resection	46,107		58													Aps
Baumgarten et al. 2021 [60]	Germany			Resection	420		56		1 to 3	Yes	MRI	Binary			None			Pre, Aps	Pre	
Blum et al. 2023 [71]	Denmark	2016–2022		Resection	38	23	67	None	1 to 3	None		Binary						Pre		
Bogdanovic et al. 2023 [82]	Serbia	2017–2019		Resection	333	224	56		1 to 3	None	MRI	Threshold	Edge* >1 cm		Yes	Yes	78 (SD 43)	Pre, Eps, Lps	Pre, Eps, Lps	
Brokinkel et al. 2021 [93]	Germany	1991–2018		Resection	405		57	Some	1 to 3	Yes	MRI	Volume		No preop		Yes		Aps		
Cai et al. 2022 [98]	China	2015–2021		Resection	517	357			1 to 3	None	MRI	Threshold	Axial Ø >1 cm	No preop	Yes	Yes		Eps	Eps	
Chaichana et al. 2013 [99]	USA	1996–2006		Resection	626		53	None	1	None	MRI	Binary			Yes	Yes		Pre	Pre	
Chen et al. 2017 [28]	USA	1991–2014	Wu[89]	Resection	843		56	None	1 to 3	None	MRI	Threshold	Edge* >1 cm		Yes			Pre, Eps, Aps	Pre, Eps	
Conti et al. 2016 [29]	Italy	2007–2014		SRS	229	145	59	None	1 or 2		MRI						60			SRS
de Vries et al. 1993 [30]	Germany			Resection	51				1 to 3	Yes	CT	Binary				Yes		Unc		
Ding et al. 2013 [31]	USA	1991–2006		SRS	49		57	None	1	None	MRI	Binary			Yes			SRS		
Ersoy et al. 2020 [32]	Germany	2015–2017		Resection	218				1 to 3		MRI	Threshold	? >1 cm							Eps, Aps
Frati et al. 2022 [33]	Italy	2016–2020		Resection	216	154	60			Yes	MRI	Threshold	cm^3, index > 1					Pre, Aps		
Gadot et al. 2021 [34]	USA	2008–2020		Resection	57		57		1 to 3	None	MRI	Binary		All preop	NA	Yes	17 (3–30)	Lps, POS		
Goertz et al. 2018 [35]	Germany	2004–2017	Goertz[36]	Resection	729				1 to 3											Pre
Goertz et al. 2023 [36]	Germany	2009–2017	Goertz[35]	Resection	44	36	60	None	1 to 3	All PF	MRI	Binary								Aps
Güngör et al. 2019 [37]	Turkey	1986–2018		Resection	21	16	43	None	1 or 2	None	CT or MRI	Binary						Pre, Eps		
Gupte et al. 2021 [39]	USA			Resection	356		58	None	1 to 3	Yes	MRI	Binary			Yes			Pre, Aps	Pre	
Hamasaki et al. 2012 [40]	Japan	1968–2011		Resection	100	65		None	1	None	MRI	Binary		recurrent preop	NA	Yes		Pre	Pre	
Hess et al. 2019 [41]	Germany	1991–2015	Hinrichs[42]	Resection	175	108	60	Some	1 to 3	Yes	MRI	Volume		No	None			Pre (d)		Aps
Hinrichs et al. 2023 [42]**	Germany	1991–2018	Hess[41]	Resection	499		58	Some	1 to 3	Yes	MRI	Volume			None			Pre	Pre	
Howng et al. 1992 [43]	Taiwan	1983–1989		Resection	87	55	50	Some		Yes	CT or MRI	Binary			None			Pre		
Hwang & Joo et al. 2019 [44]	Korea	2003–2014	Hwang[45]	Resection	303	215	54	Some	1 to 3	None	MRI	Binary			Yes	Yes	49 (1–137)	Lps	Pre, Lps	
Hwang & Kim et al. 2019 [45]	Korea	2009–2016	Hwang[44]	SRS	133	95	59	Some	1	None	MRI	Binary			None			SRS		
Im et al. 2001 [46]	Korea	1981–1999		Resection	10	5	8	All		Yes	CT or MRI	Binary						Pre, Aps (p)		
Islim et al. 2018 [47]	England	2010–2015		Resection	283	214	58		1 to 3	Yes	MRI	Threshold	Index > 0–5%		Yes	Yes		Pre, Aps	Pre	
Jung et al. 2022 [48]	Korea	2019–2020		SRS	127	108	60			None	MRI	Binary		No	None		10			SRS
Kawaguchi et al. 1996 [50]**	Japan	1976–1994		Resection	61		57	None		None	CT	Area						Pre	Pre	
Kemerdere et al. 2019 [51]	Turkey	2010–2017		Resection	63	45	52	None	1 or 2	None	MRI	Binary			Yes		47 (12–96)	Pre	Pre, Lps	
Kim et al. 2019 [52]	Korea	2013–2016		Resection	26	18	59	None	1 to 3	Yes	MRI	Threshold	Edge* >1 cm					Pre		
Kirn et al. 1998 [53]				Resection	66					Yes	MRI	Area		Yes	None					Unc
Kollova et al. 2007 [54]	Czechia	1992–1999		SRS	368		57	None	1	Yes	CT or MRI						68 (24–126)		SRS	
Kuhn et al. 2014 [55]	USA	1999–2011		SRS	194	134	62	Some	1 to 3	Yes	MRI	Binary		No pre SRS				SRS		
Lazzarin et al. 2022 [56]	Italy			Craniotomy																Eps
Le et al. 2023 [57]	Vietnam	2020–2022		Resection	15						MRI	Threshold	> 1 cm							Aps
Li & Wang et al. 2020 [59]	China	2011–2012		Resection	772	537	50	Some	1 to 3	None	MRI	Threshold	Edge* >1 cm		Yes			Pre, Eps, Aps	Pre, Eps	
Li & Zheng et al. 2021 [58]	China	2008–2018		Resection	117		52	None	1 to 3		MRI	Threshold	Worse postop					POS		
Lieu et al. 2000 [61]	Taiwan	1982–1997		Resection	214		50	1.4%		Yes	CT	Threshold	Marginal			Yes		Pre, Aps	Pre	
Lobato et al. 1996 [62]	Spain	1974–1999		Resection	400	282	54	Some		Yes	CT	Binary		No	None					Pre
Loewenstern et al. 2019 [63]	USA	2002–2016		Resection	112	82	71	None	1 to 3	None	MRI	Volume		Yes	None					Pre
Maeder et al. 1984 [64]	Switzerland			Resection	80	43	53			Yes	CT	Binary						Pre		
Markovic et al. 2013 [65]	Serbia	2009–2011		Resection	78		61	None		None	CT or MRI	Binary				Yes		Pre		
McKevitt et al. 2023 [66]	USA	2012–2022		Resection	113	81	59		1 to 3	Yes	MRI	Bin/Vol		No preop	Yes	Yes		Aps		
Mohme et al. 2016 [67]	Germany	1988–2015		Resection	117		59	None	1	None	CT or MRI	Threshold	~ index > 1		None			Pre		
Morsy et al. 2019 [68]	Egypt			Resection	40	28	58	None		None	CT or MRI	Binary			Yes	Yes		Pre, POS	Pre	
Nassar et al. 2022 [70]	Ukraine	2007–2018	Nassar[69]	Resection	244	165	54	None	1 to 3	None	MRI	Binary			None		3 exactly	Pre, LPS	Pre	
Nassar et al. 2022 [69]	Ukraine	2007–2020	Nassar[70]	Resection	65	49	54	Some	1 to 3	None	MRI	Binary						Pre (d)	Pre	
Panagopoulos et al. 2008 [72]	Brazil	1999–2005	Simis[79]	Resection	25	11	53	4%	1 to 3	Yes	MRI	Threshold	Slight halo					Unc		
Patil et al. 2008 [73]	USA	2001–2006		SRS	102		60	None		None	CT or MRI						21 (6–77)			SRS
Pauletto et al. 2023 [74]	Italy	2016–2020		Resection	342		62	none	1 to 3	Yes	MRI	Binary				Yes		Pre	Pre	
Rajab et al. 2022 [75]	Syria	2017–2021		Resection	97	64		6%	1 to 3	None		Binary						Pre, Aps		
Salpietro et al. 1997 [76]				Resection	66					None	CT or MRI	Threshold	Finger like	No	None					Pre
Schneider et al. 2019 [77]	Germany	2009–2017	Wach[86]	Resection	187	121	60	None	1 to 3	None	MRI	Threshold	Axial Ø >1 cm	All preop	NA	Yes	12 exactly	Lps		
Seyedi et al. 2018 [78]	Denmark	2007–2015		Resection	295	197		None	1 to 3	None	MRI	Binary			None			Pre, Aps	Pre	
Simis et al. 2008 [79]	Brazil	1993–2006	Panag[72]	Resection	61	40	57	None	1	None	MRI	Threshold	**Slice > 2 cm	No	None					Pre
Singh et al. 2023 [80]	India	2007–2020		Resection	333	157	44		1 to 3		MRI	Binary		All preop	NA	Yes		Aps		
Skardelly et al. 2017 [81]	Germany	2007–2012		Resection	634	458	58	None	1 to 3	Yes	MRI	Binary		No	None					Unc
Stevens et al. 1983 [83]	England			Resection	160					None	CT	Threshold	Moderate			Yes		Pre		
Teske et al. 2024 [84]	Germany	2013–2023		Resection	95	63	60	None	2 or 3	None	MRI	Bin/Vol			Yes	Yes	21 (1–128)	Pre, Eps, Lps	Pre	
Tsuji et al. 1993 [85]	Japan	1990–1992		Resection	19		53	None		Yes	CT	Binary			Yes			Pre, Aps, POS		
Wach et al. 2022 [86]	Germany	2009–2022	Schn[77]	Resection	330		61		1 or 2	Yes	MRI	Binary						Pre	Pre	
Wang et al. 2018 [87]	Taiwan	2001–2009		Resection	102	57	57		2 or 3	Yes	CT or MRI	Binary			Yes		78 (5–195)	Pre, Eps, Lps	Pre, Eps, Lps	
Wirsching et al. 2016 [88]	Switzerland	2000–2013		Resection	692		57	None	1 to 3	Yes	CT or MRI	Binary			Yes	Yes	67 (CI 63–72)	Pre, Lps	Pre, Lps	
Wu et al. 2017 [89]	USA	1990–2005	Chen[28]	Resection	283	186	59	Some	1 to 3	Yes									Pre	
Xiao et al. 2021 [90]	China	2017–2019		Resection	136	35	54				MRI	Threshold	Worse postop					POS		
Xu et al. 2021 [91]	China	2014–2016		Resection	260	172			1 to 3		MRI	Threshold	cm^{3} Index > 4		None			Eps	Eps	
Xue et al. 2018 [92]	Sweden	2006–2008		Resection	113	94	53	None	1 or 2	Yes	MRI	Threshold	Gross oedema	No	None					Aps
Yang et al. 2020 [94]	China	2016–2018		Resection	186	134		None	1 to 3	None	CT or MRI	Binary			Yes			Aps		
Zachenhofer et al. 2006 [95]	Austria	1992–1995		SRS	36	30	59	None	1 to 3	None							(70–133)			SRS
Zhang et al. 2020 [97]	China	2014–2018		Resection	318	222		None	1 to 3	Yes		Threshold	? >1 cm		Yes		27 (6–56)	Lps	Lps	
Zhang et al. 2015 [96]	China	2000–2010		Resection	209	134	68	None		Yes	CT or MRI	Binary		No preop	Yes				Eps	

*Age as mean or median years. **compared oedema area or volume in patients with and without seizure

*n* = number of participants, *Blank *data not provided, *Pre *preoperative seizure, *Eps *early postoperative seizure, *Lps *late postoperative seizure, *Aps *any postoperative seizure, *SRS *post radiosurgery seizure and oedema, *POS *postop oedema and seizure, *Unc *unclear whether seizure was pre or postop, *(d) *duplicate and excluded, *(p) *paediatric and excluded

### Preoperative oedema and preoperative seizures

**Fig. 3:**
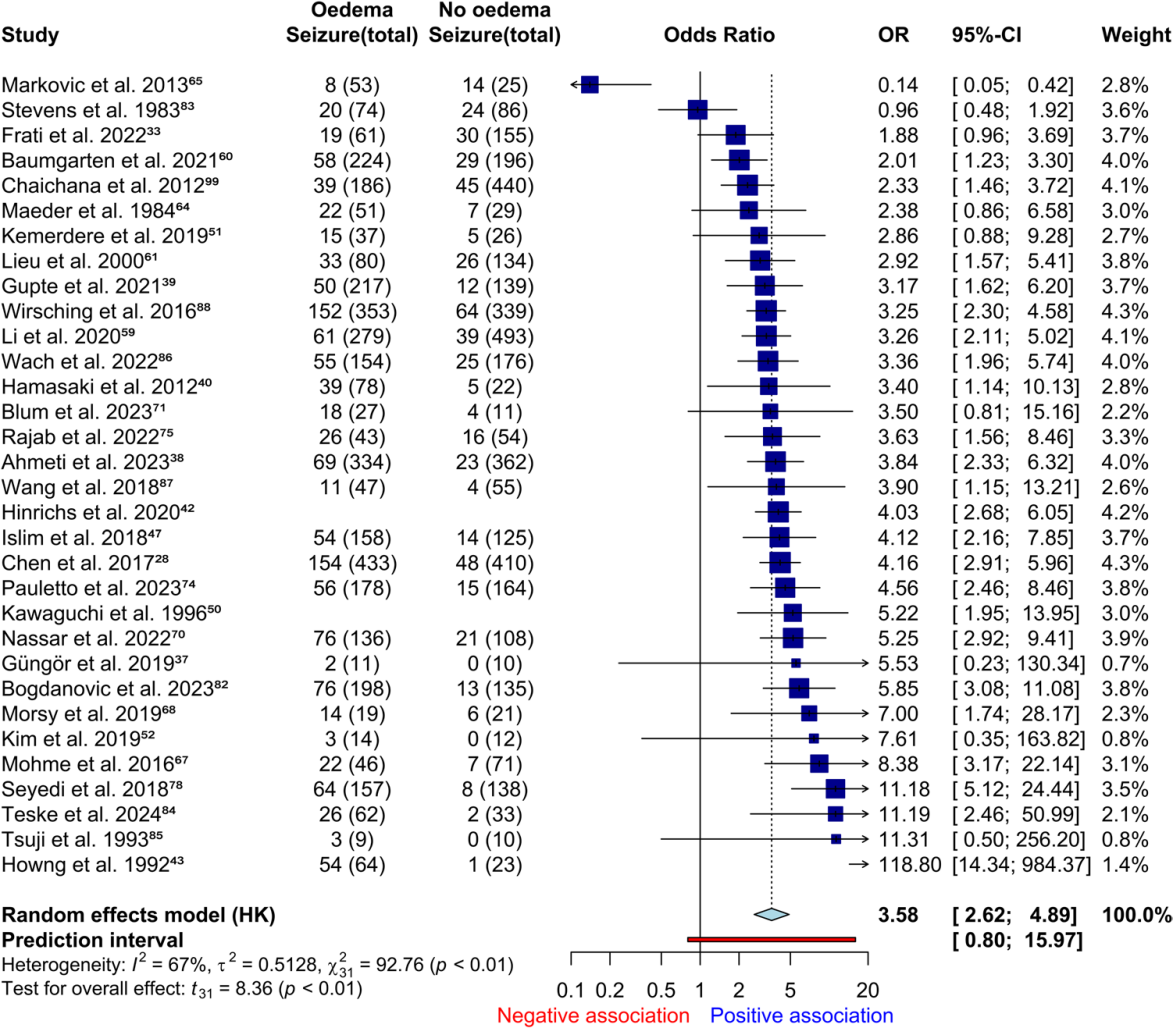
Forest plot of preoperative oedema and preoperative seizure, unadjusted with outliers

In our meta-analysis, preoperative oedema significantly increased the odds of preoperative seizure (*k* = 32, *n* = 8,345, *OR* 3.6, 95% *CI* = 2.6–4.9, *I*2 = 67%, Fig. 3). Only 13% of patients without oedema had seizure, whilst 34% with oedema had seizure. Heterogeneity was moderate, rectified by removal of outlying studies (*k* = 28, *n* = 7,725, *OR* 3.5, 95% *CI* = 3.1–4.0, *I*2 = 0%, Online Resource 4, GRADE: high). In our covariate review preoperative oedema was a significant predictor of preoperative seizure in univariable (95%, *k* = 21) and multivariable analysis (81%, *k* = 16) (Online Resource 5 and 6). Stevens et al. proportioned seizure semiology in patients with oedema: focal − 50%, grand mal – 26% [83]. Chaichana et al. found oedema to be unrelated to uncontrolled preoperative seizures [99]. Seven additional studies (not eligible for meta-analysis or covariate review) described relationships between preoperative oedema and preoperative seizures with mixed results (Online Resource 7).

### Preoperative oedema and postoperative seizures

**Fig. 4:**
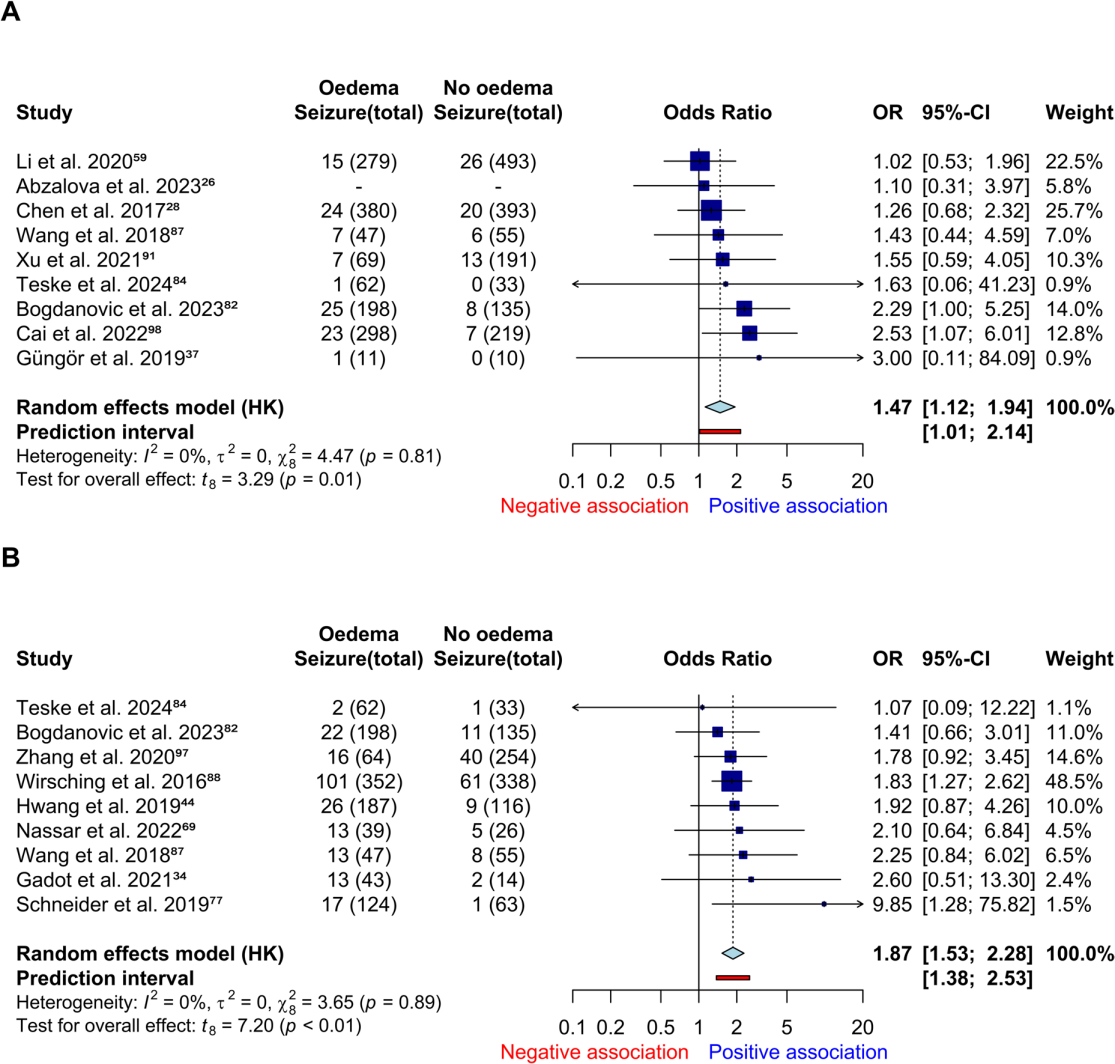
Forest plot of: **A** preoperative oedema and early postoperative seizure, **B** preoperative oedema and late postoperative seizure

There were 28 eligible studies for meta-analysis of preoperative oedema and postoperative seizure: nine early (< 1 week), nine late (> 1 week) and 15 unclear. Oedema was associated with early postoperative seizures (*k* = 9, *n* = 2,929, *OR* 1.5, 95% *CI* = 1.1–1.9, *I*2 = 0%, Fig. 4A, GRADE: moderate). There were no outliers. Proportions with seizure increase from 5 to 8% when oedema is seen. Two additional studies were suitable for narrative review (Online Resource 7) with contrasting conclusions. Oedema was significantly associated with late postoperative seizures (*k* = 9, *n* = 2,150, *OR* 1.9, 95% *CI* = 1.5–2.2, *I*2 = 0%, Fig. 4B, GRADE: moderate). Proportions with seizure increase from 13 to 20% when oedema was present. There were no outliers. We pooled postoperative seizure studies and selected unique subsets from each study (Online Resource 8). Preoperative oedema increased risk of postoperative seizure (*k* = 32, *n* = 8,181, *OR* 1.6, 95% *CI* = 1.4–2.0, *I*2 = 65%). Postoperative seizure proportions increase from 10 to 18% with preoperative oedema. Outlier removal results in low heterogeneity (*k* = 31, *n* = 7,776, *OR* 1.8, 95% *CI* = 1.5–2.1, *I*2 = 10%, GRADE: moderate, Online Resource 4). Seizures could have occurred any time within postoperative follow up (one to 286 months, Online Resource 8) but two studies specified seizure outcome at 3 or 12 months postoperatively [69, 77]. In covariate review, oedema was seldom a predictor for seizures in univariable analyses (Early: 14% of seven studies, Late: 20% of five studies, All: 44% of 16, Online Resource 5) and multivariable analyses (Early: 33% of three studies, Late: 33% of three studies, All: 22% of nine, Online Resource 6). There was no association between preoperative oedema and refractory epilepsy in one study [82].

### Radiotherapy and seizures

**Fig. 5:**
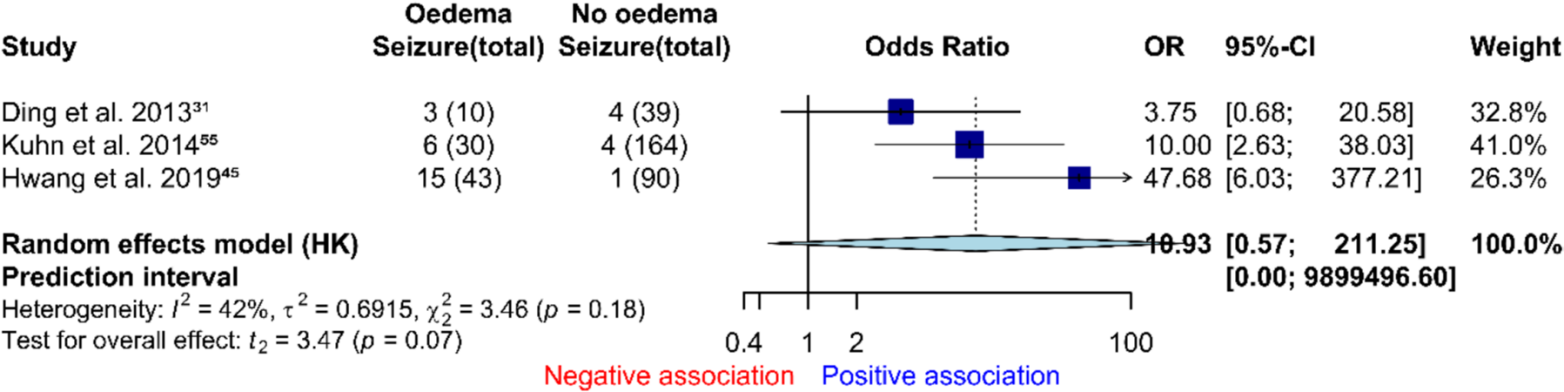
Forest plot of post radiosurgery oedema and seizure

Eight studies reported oedema and seizure following radiosurgery (Online Resource 9). Post-treatment oedema occurred in 15%, and 4% had post-treatment oedema and seizure. It is unclear whether oedema precedes seizures in these reports. Two studies noted oedema occurring an average of seven months after CyberKnife treatment [29, 73]. In our meta-analysis, post-radiosurgery oedema was not associated with post-radiosurgery seizure (*k* = 3, *n* = 376, *OR* 10.9, 95% *CI* = 0.6–211.3, *I*2 = 42%, GRADE: very low, Fig. 5). Proportions of seizure in patients with post treatment oedema was 6% compared to 2% without.

### Other associations between oedema and seizures

#### Paediatric

Im et al. studied 10 children (median age eight years) with operated meningioma [46]. They found no association between preoperative oedema and preoperative seizure (*OR* 1.0, 95% *CI* 0.1–12.6) or postoperative seizure (*OR* 0.4, 95% *CI* 0.1–12.6) [46]. Some studies did include paediatric patients, but only three specified proportions (range 1.4–6.0%, Table 2), so Im et al. was excluded from the meta-analyses. For sensitivity analysis we repeated our meta-analyses with Im et al. Results were similar for preoperative oedema and seizure (*k* = 33, *n* = 8,355, *OR* 3.5, 95% *CI* = 2.6–4.8, *I*2 = 66%). Results were also similar for preoperative oedema and unknown postoperative seizure with Im et al. (*k* = 16, *n* = 4,639, *OR* 1.8, 95% *CI* = 1.5–2.3, *I*2 = 79%) and without (*k* = 15, *n* = 4,629, *OR* 1.9, 95% *CI* = 1.5–2.4, *I*2 = 80).

#### Postoperative oedema

Six studies noted postoperative oedema. Preoperative seizure was not significantly associated with new/worsening postoperative oedema, but postoperative seizures and postoperative oedema were associated (Online Resource 4).

### Subgroup analysis and meta-regression

We performed subgroup analysis (minus outliers) and meta-regression for preoperative oedema and preoperative or (any) postoperative seizure (Online Resources 10 to 15). For preoperative seizure there was no difference by continent of study, inclusion of infratentorial tumour, imaging modality used for oedema, oedema measurement, or use of seizure definition. Very high risk of bias was associated with an inflated *OR* and subgroup difference (Online Resource 10), but there was no significant difference in meta-regression (Online Resource 12 and 13). We subset studies of postoperative seizure by preoperative seizure status (Online Resource 8). For preoperative oedema and postoperative seizure, there were no differences with risk of bias, preoperative seizure status, postoperative seizure status (early versus late), infratentorial tumour inclusion, continent, imaging modality, oedema measurement, and with prophylactic ASM use (any proportion) in seizure naïve patients.

### Publication bias

**Fig. 6:**
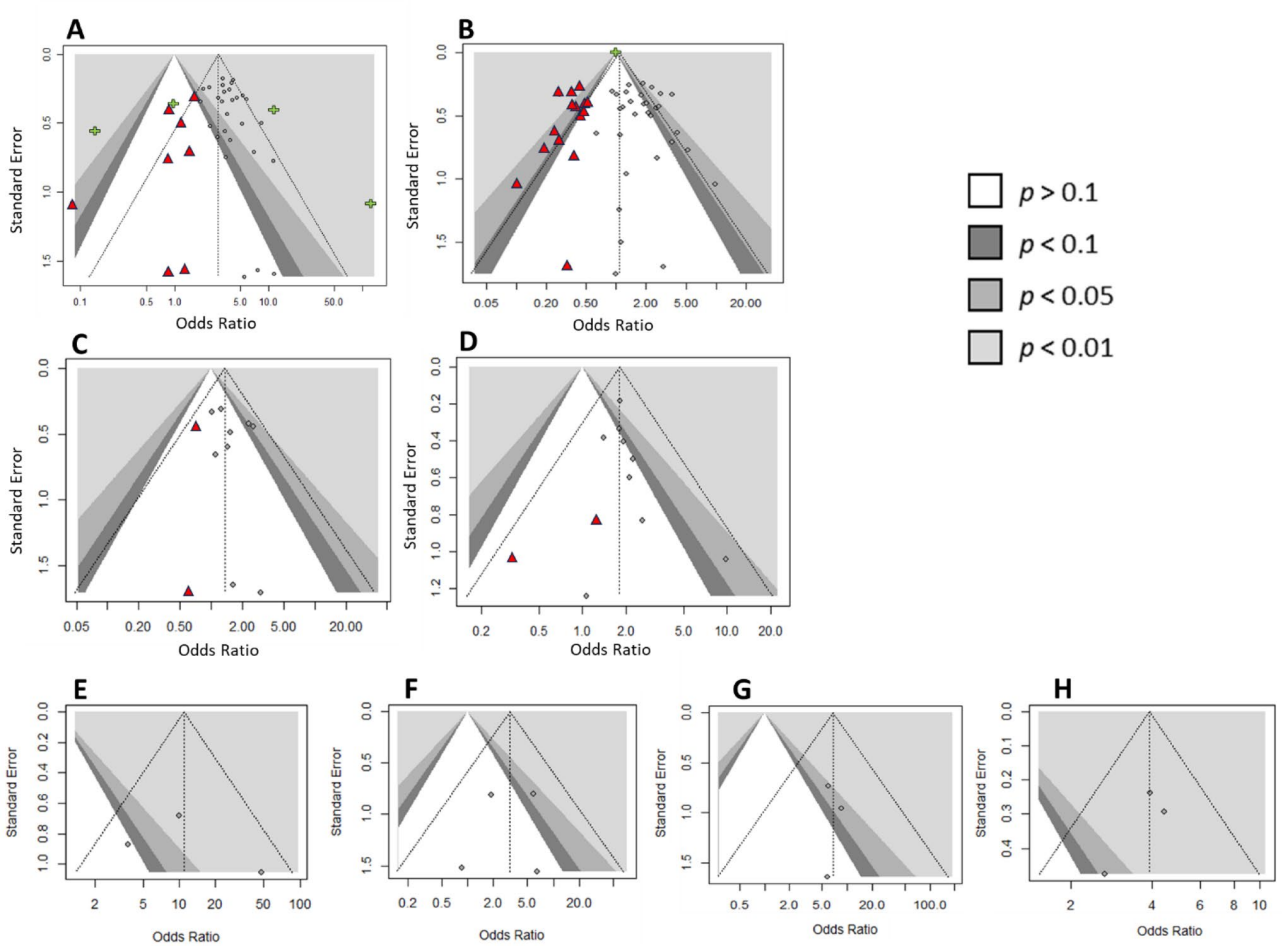
Funnel plots for meta-analyses of: **A** Preoperative oedema and preoperative seizure; **B** Preoperative oedema and postoperative seizure; **C** Preoperative oedema and early postoperative seizure; **D** Preoperative oedema and late postoperative seizure; **E** Post radiosurgery oedema and seizures; **F** Preoperative seizure and postoperative oedema; **G** Postoperative seizure and postoperative oedema and (**H**) Preoperative oedema and seizure (adjusted). Grey circle denotes study, green cross denotes outliers, and red triangle denotes simulated study using trim and fill

Funnel plots and Egger’s test suggested publication bias for preoperative oedema and all postoperative seizures (Fig. 6B, Online Resource 4). This was resolved on outlier removal as demonstrated by repeat funnel plots, Egger’s test and *p*-curve analysis (Online Resource 4 and 16). There was no evidence of publication bias for other analyses.

### Covariate review

We noted all non-oedema seizure predictors in univariable and multivariable tests (Online Resource 5 and 6). Factors associated with preoperative seizures included falcine (100%, *k =* 3) or parasagittal locations (60%, *k* = 5), brain invasion (60%, *k =* 5) and oedema (95%, *k =* 21). Negative associations included headache (83%, *k* = 6), preoperative deficit (71%, *k* = 7) and skull base tumours (67%, *k* = 9). On multivariable analyses only oedema (81%, *k* = 16) was consistently reported as a positive and headache (100%, *k =* 6) a negative predictor.

For any postoperative seizure, preoperative seizures (85%, *k =* 13), postoperative deficit (67%, *k* = 6) and tumour recurrence (67%, *k =* 9) were significant. In multivariable testing, only presence of complications (75%, *k* = 8) was. Univariate positive predictors of early postoperative seizure included motor cortex proximity (100%, *k* = 2), preoperative seizures (80%, *k* = 5), postoperative deficit (100%, *k =* 4) and surgical complications (75%, *k* = 4). In multivariable analyses, motor cortex proximity (100%, *k* = 2) and surgical complications (100%, *k* = 3) remained significant. For late postoperative seizures univariable predictors included convexity location (75%, *k* = 4), preoperative seizures (100%, *k* = 4) and tumour recurrence (80%, *k* = 5). In multivariable analysis, preoperative seizures (66%, *k* = 6) and recurrent tumour (60%, *k* = 5) were significant.

Pre-radiosurgery oedema was a univariable predictor of post-treatment seizure in Kollova et al. [54]. In Hwang et al.. it was a univariable but not multivariable predictor, but post-treatment oedema was associated with post-treatment seizure in univariable and multivariable analysis [45].

### Adjusted meta-analysis

We performed an adjusted meta-analysis for preoperative oedema and preoperative seizure. Preoperative oedema, headache and gender were selected as core predictors from our narrative and covariate review, and the unadjusted meta-analysis by Englot et al. [2]. Three studies in our meta-analysis provided suitable multivariable results with these core predictors (Online Resource 17). They also corrected for tumour size, and two corrected for non-skullbase location. None had high risk of bias on ROBINS-E. Preoperative oedema remained a significant predictor of preoperative seizure when adjusting for other predictors (*k* = 3, *n* = 2,241, *OR* 3.9, 95% *CI* = 2.4–6.3, *I*2 = 0%, Online Resource 4). There were no outliers or evidence of publication bias (Fig. 6H, Online Resource 4 and 16). There was insufficient data to perform an adjusted analysis of postoperative seizures accounting for any “core” postoperative variable: proximity to the motor cortex, postoperative deficit, preoperative seizure, or surgical complication.

## Discussion

We provide high GRADE evidence that preoperative oedema is a prognostic factor for preoperative seizures (Table 3). Once outliers are removed there is low heterogeneity and all studies show a positive association. Preoperative oedema increases proportions with seizure from 12 to 34%. Our exploratory covariate review and adjusted meta-analysis also suggest that oedema is a key prognostic factor even when correcting for headache and gender (Table 3). This is a novel finding. The literature was unclear on whether oedema preceded seizure; clarification of this would be of interest. Another unadjusted meta-analysis by Englot et al. also found a significant association between preoperative oedema and seizure (*K* = 8, *n* = 1,095, *OR* 7.5, 95% *CI* 6.1–7.5) [2]. The *OR* appears high for the data presented in their forest plot and may be erroneous [2]. We were unable to reproduce their findings by meta-analysing the studies in their analysis (Online Resource 18), which revealed an *OR* more similar to ours (Fig. 2). In contrast with our covariate review, Englot et al. also identified age as a negative predictor for preoperative seizures, but a meta-analysis would be more sensitive in identifying a true association [2]. Adjusted meta-analysis of preoperative seizure risk factors, such as gender, age and headache, would be of interest.

**Table 3:** Summary of findings table

Outcome		Prognostic factor	*n* (k)	Seizure proportion		Odds ratio (95% CI)	GRADE of evidence	Justification	Plain text summary
				Without oedema	With oedema				
**Preoperative seizure**^**a**^		**Preoperative oedema**	7,725 (28)	12%	34%	3.5 (3.1–4.0) 3.9 (adj^b^) (2.4–6.3)	⊕⊕⊕⊕ High	Without outliers, all studies showed a positive association (meta-analysis), no studies rated “Very high” for risk of bias and subgroup analysis appeared similar for “Low” to “High” risk of bias. Without outliers there was low heterogeneity across measures and no evidence of publication bias. Preoperative oedema is consistently a significant predictor of preoperative seizure in multivariable tests and when controlling for other factors in an adjusted analysis.	In surgical populations, preoperative oedema increases the odds of preoperative seizure.
**Postoperative seizure**	**Any**^**a**^ (range 1 day to 17 years)	**Preoperative oedema**	7,776 (31)	10%	18%	1.8 (1.5–2.0)	⊕⊕⊕◯ Moderate	Without outliers some studies were rated as “Very high” for risk of bias in confounding and measurement of oedema but there was no significant difference in subgroups by risk of bias, heterogeneity measures were low and there was no evidence of publication bias. Oedema was rarely a significant predictor of postoperative seizure in multivariable tests.	Preoperative oedema increases risk of postoperative seizures at all time points. Other independent factors might be more influential and will be different for early and late postoperative seizures.
	**Early** (within 7 days)	**Preoperative oedema**	2,929 (9)	5%	8%	1.5 (1.2–1.9)	⊕⊕⊕◯ Moderate	Effect direction was positive in all studies (meta-analysis). While some studies had “High” or “Very high” risk of bias due to confounding, measurement of oedema and post-exposure interventions, most studies had “Low” or “Some” risk of bias. There was low heterogeneity across measures and no evidence of publication bias. Oedema was rarely a significant predictor of early postoperative seizure in multivariable tests.	
	**Late** (range 1 month to 17 years)	**Preoperative oedema**	2,150 (9)	13%	20%	1.9 (1.5–2.2)	⊕⊕⊕◯ Moderate	Effect direction was positive in all studies (meta-analysis). While some studies had “High” or “Very high” risk of bias due to confounding, measurement of oedema and post-exposure interventions, most studies had “Low” or “Some” risk of bias. There was low heterogeneity across measures and no evidence of publication bias. Oedema was rarely a significant predictor of early postoperative seizure in multivariable tests.	
**Post radiosurgery**^**c**^ **seizure**		**Post radiosurgery oedema**	376 (3)	3%	29%	10.9 (0.6–211.3)	⊕◯◯◯ Very low	Direction of effect was positive in all studies (meta-analysis). Small sample size. Moderate heterogeneity. Wide confidence intervals. Most studies had high risk of bias. There was no evidence of publication bias	Radiosurgery may lead to oedema and seizures, further research is warranted.

^a^ Without outliers

^b^ Adjusted (adj) for headache and gender among other variables (supplementary resource 16), all other results in this table are unadjusted

^c^ Gamma knife, dose range 10–20 gy, proportions with prior surgery range from 16–80%, follow up maximum 12 years

There is moderate GRADE evidence that preoperative oedema predicts early and late postoperative seizures (Table 3). All studies in the meta-analysis demonstrated a positive association and heterogeneity was low. For early postoperative seizures risks increased from 5 to 8% when oedema was present, and for late postoperative seizures it increases from 13 to 20%. Beyond one week, it was not possible to provide more discreet postoperative seizure timings. We can infer from the meningioma literature that most postoperative seizures occur within a year, and that 70–90% of patients are seizure free within a few years (Online Resource 3). Our meta-analysis agrees with the meta-analysis of Ghazou et al. who found preoperative oedema to be a predictor of late postoperative seizures (*k* = 5, *n* = 1,721, *OR* 2.0, *CI* 1.5 − 2.6) [12]. However in contrast, Ghazou et al. found a positive but insignificant association between oedema and early postoperative seizure (*k* = 4, *n* = 2,164, *OR* 1.4, 95% *CI* 0.96–2.00) and this is likely due to their reduced sample size; our analysis of early postoperative seizures had 11 studies and 2,929 participants with a very similar *OR* of 1.5 [12].

This is the first meta-analysis in meningioma and seizure to use subgrouping and meta-regression. We did not identify any study level characteristic that significantly modified the relationship between preoperative oedema and preoperative or postoperative seizures, this includes continent of study which suggests similar findings are seen across ethnic backgrounds. Furthermore, there was no difference in postoperative seizure risk (due to oedema) by presence of preoperative seizure; perhaps this is due to treatment of oedema or seizure. Regarding prophylactic ASM use in seizure naive patients and postoperative seizure risk (due to oedema), no difference was found but proportions with prophylactic ASM did vary across studies so findings are limited. We suspect other factors might be more important for postoperative seizures, such as tumour location or tumour recurrence and surgical complications, but we were unable to control for these factors in our adjusted meta-analyses. 

This is the first meta-analysis of post-radiosurgery oedema and seizure, and of postoperative oedema. There are too few studies to comment conclusively on these populations, but it does appear that post-treatment oedema and seizure may be correlated, and that necrosis could be implicated in post-radiotherapy oedema [48]. No studies reported on oedema and seizure risk in conservatively managed meningioma.

For healthcare providers this meta-analysis quantifies the effect of oedema on seizure risk pre and postoperatively. This will aid counselling and guide monitoring but will not inform use of prophylactic medications. This will be addressed in randomised controlled trials and oedema should be used to stratify seizure risk in these studies [9].

There was little discussion of oedema and seizure frequency, severity or semiology. One study did not find any differences in preoperative seizure control when oedema was present, and another found no link with refractory postoperative seizure [82, 99]. Better identification of patients at risk of refractory epilepsy could highlight those that would benefit from epilepsy surgery workup in future.

### Limitations

Despite checks to minimise data validation errors, there is still a risk of errors. Google scholar is discouraged in systematic reviews due to issues with storage and reproducibility [100]. It does, however, serve as a useful adjunct; it identified 12 further studies eligible for meta-analysis and three for narrative review.

Investigation of oedema and seizure risk was not the primary aim of the studies in this meta-analysis; most looked for seizure risk factors more generally. As a result, many studies had high risks of bias due to issues with oedema measurement or confounding factors on ROBINS-E. This was mitigated in the analysis of preoperative seizure by removal of outliers which also had very high risk of bias. For measurement of oedema, our subgroup and regression analyses suggest that reports with differing imaging modalities or oedema definitions had similar results. Furthermore, as we were mostly using unadjusted effect sizes, the issue of accounting for confounding factors is less problematic.

The categories in our subgroup and regression analyses may not have been distinct enough to detect differences for prophylactic ASM and infratentorial categories. While we were able to perform an adjusted meta-analysis for preoperative seizures, there was insufficient data in the literature for postoperative seizures. Unadjusted effect sizes are inherently limited as they do not consider the effects of other factors.

The findings from our covariate review are exploratory and descriptive, it is not possible to confirm the number of patients included for each variable in each analysis, and no statistical analysis was performed. The aim of the covariate review is to aid direction of future meta-analysis on seizures in meningioma.

There is a limited literature base for oedema and seizure risk in radiosurgery, conservatively managed meningioma and in paediatric populations which need further exploration when more studies are available. Some authors suggest that seizures are more common in paediatric meningioma [2, 46].

## Conclusion

This is the first meta-analysis in meningioma, oedema and seizures to use subgrouping, meta-regression and adjusted analysis. Preoperative oedema is a key adverse prognostic factor for the development of preoperative seizures in meningioma patients. Preoperative oedema signals a modest increased risk of early and late postoperative seizure but other factors might be more important. We were unable to find any study level characteristics that altered risk of pre or postoperative seizure due to oedema. This is the first meta-analysis of seizure risk due to post-radiosurgery oedema which revealed a positive but insignificant association, further research is warranted.

## Supplementary information

Below is the link to the electronic supplementary material.

ESM 1 (DOCX 922 KB)

## Data Availability

Data is provided within the manuscript or supplementary information files.
