# Supplementary material for: Oedema as a prognostic factor for seizures in meningioma - a systematic review and meta-analysis
Source: Neurosurg Rev. 2025 Feb 19;48(1):249. doi: 10.1007/s10143-025-03416-1 (PMC11839703; doi:10.1007/s10143-025-03416-1)
Supplement: Supplementary file 1 — (DOCX 922 KB) [file 10143_2025_3416_MOESM1_ESM.docx]

# Supplementary online resources

Article title: “Oedema as a prognostic factor for seizures in meningioma - a systematic review and meta-analysis”

Authors: Matthew John Tanti, Sarah Nevitt, Molly Yeo, William Bolton, Paul Chumas, Ryan Mathew, Melissa Jane Maguire

Journal: Neurosurgical review

Corresponding email address: [m.tanti@nhs.next](mailto:m.tanti@nhs.next)

Note: References from the supplementary information are separate to the main text

### Online Resource 1: Database search strategy

| Database | Search term | Other criteria |
| --- | --- | --- |
| OVID | “(meningioma OR meningiomas) AND (epilepsy OR epileptic OR seizure OR seizures) AND (oedema OR edema)” | All fields |
| Scopus | “(meningioma OR meningiomas) AND (epilepsy OR epileptic OR seizure OR seizures) AND (oedema OR edema)” | All fields |
| Pubmed (medline) | “meningioma* AND (epilep* OR seizure*) AND (oedema OR edema)” | All fields |
| Web of science | “meningioma* AND (epilep* OR seizure*) AND (oedema OR edema)” | All fields |
| ClinicalTrials.gov | Condition: “meningioma OR meningiomas” Other terms: “epilepsy OR epileptic OR seizure OR seizures OR oedema OR edema” |  |
| Google scholar | “meningioma, seizure OR epilepsy, edema OR oedema” | Restricted to first 200 articles in 2023 and in 2024 |

### Online Resource 2: R programs used

| **R Program** | **Use** | **Credit to** |
| --- | --- | --- |
| meta | Pooled effect size  Subgroup analysis  Forest plot  Funnel plot | Balduzzi *et al.*^1^ |
| dmetar | Eggers test  *p* curve  Multimodel inference | Harrer *et al.*^2^ |
| metafor | Regression  Permutation test | Viechtbauer^3^ |
| PerformanceAnalytics | Collinearity | Peterson & Carl^4^ |
| tidyverse | Generic | Wickham *et al.*^5^ |
| mice | Multiple imputation | van Buuren *et al.*^6^ |
| poibin | Generic | Hong^7^ |
| stringr | Generic | Wickham^8^ |
| naniar | Missing data | Tierney & Cook^9^ |

### Online Resource 3: Summary of seizure semiology/definitions and prophylactic ASM use

| **Study** | **Seizure semiology/definition** | **Study** | **Prophylactic ASM** |
| --- | --- | --- | --- |
| Abzalova *et al*. 2023^10^ | New early postoperative generalised tonic clonic seizures occurred in 15%. | Abzalova *et al*. 2023^10^ | Patients without preoperative and pre-seizure ASM were excluded. |
| Bogdanovic *et al*. 2023^11^ | Any semiology included. ILAE definition of epilepsy used and 32% of those with postoperative seizures had refractory epilepsy. Presence of oedema was not associated with refractory postoperative epilepsy. | Bogdanovic *et al*. 2023^11^ | 27% of patients had preoperative seizures, and 81% of all patients had preoperative ASM. |
| Brokinkel *et al*. 2021^12^ | New postoperative seizures occurred in 69 individuals (9%), of which 51% were focal and 32% were generalised. | Cai *et al*. 2022^13^ | All had preoperative prophylactic ASM. |
| Cai *et al*. 2022^13^ | Seizure semiology was collected but not presented. Of 30 with early postoperative seizures, 17 had less than 2 seizures, 10 had more than 2 seizures, and 3 developed status epilepticus. | Chaichana *et al*. 2013^14^ | Patients usually given prophylactic ASM just before surgery. |
| Chaichana *et al*. 2013^15^ | In patients with preoperative seizures, 65% had focal, 8% had complex partial and 35% had generalised seizures. Presence of generalised seizures was associated with lower chance of uncontrolled preoperative seizures. At 4 years postop 90% were Engel class I, 3% were class II, 0 were class III, and 7% were class IV. Patients with controlled preoperative seizures were more likely to have better postoperative seizure outcomes. | Chen *et al*. 2017^16^ | Patients were placed on prophylactic ASM for surgery and 7 days postop if tumours were frontal or temporal, if there was oedema, or if the pia was disturbed during surgery. |
| de Vries *et al*. 1993^17^ | Focal and generalised seizures were included. | Ding *et al*. 2013^18^ | 17% had seizures on presentation and 57% had pre gamma knife ASM. It is not known how many patients developed seizures after presentation and before radiosurgery. |
| Gadot *et al*. 2021^19^ | In patients with preoperative seizures, 39% had focal aware, 14% focal unaware, 35% generalised tonic clonic, 9% had non motor generalised and 4% were not classified, 42% had engel class I outcome at follow up, which was median 17 months. There was no association between semiology and engel outcome. | Gupte *et al*. 2021^20^ | 44% of patients had perioperative prophylactic ASM. |
| Hamasaki *et al*. 2012^21^ | Preoperative seizures consisted of the following semiologies: simple partial 30%, complex partial 7%, generalised 34%, partial to generalised 30%. | Hwang & Joo *et al*. 2019^22^ | 97% had prophylactic ASM at surgery and for a month after. |
| Hwang & Joo *et al*. 2019^22^ | In patients with preoperative seizure, 67% had engel class one at average follow up of 55 months. In patients without preoperative seizures 88% were engel class one (follow up unclear but entire population had follow up of 49 months. | Islim *et al*. 2018^23^ | 76% were in receipt of prophylactic ASM at the time of surgery which remained for a median of 275 days. |
| Islim *et al*. 2018^23^ | In patients with preoperative seizures, 24% had focal motor seizures, 13% focal non motor, 10% focal with impaired awareness, 53% focal to generalised. Of 17% with postoperative seizures, 35% occurred within 1 week, an additional 29% within 6 months, 10% within a year and 25% beyond a year. At 12 months of follow up, 90% of seizure naïve were seizure free compared to 79% with preoperative seizure. | Kemerdere *et al*. 2019^24^ | 12% had preoperative prophylactic ASM despite not having preoperative seizures. |
| Lieu *et al*. 2000^25^ | For preoperative seizures, 68% were generalised, 27% were partial, 3% partial with generalisation, 2% complex partial. | Li & Wang *et al*. 2020^26^ | Perioperative prophylactic ASM was given to 98% of patients and postoperative prophylactic ASM was given to 88%. |
| Markovic *et al*. 2013^27^ | Seizures were described as focal or generalised. | McKevitt *et al*. 2023^15^ | 64% of patients received perioperative prophylactic ASM for a median period of 14 days. |
| McKevitt *et al*. 2023^15^ | Of 11 patients with new postoperative seizures, 55% had generalised seizures, 36% had focal aware and one patient had a tonic aware seizure. | Morsy *et al*. 2019^28^ | All patients were loaded with phenytoin intraoperatively. |
| Morsy *et al*. 2019^28^ | Postop engel outcome was collected but not presented. | Teske *et al*. 2024^29^ | 9% received prophylactic ASM prior to surgery. |
| Pauletto *et al*. 2023^30^ | In patients with preoperative seizures, 62% had an isolated seizure. Focal seizures were present in. 38% and focal to generalised seizures in 62%. 80% of seizures were motor, 14% sensory and the remainder were cognitive or autonomic. At 24 months 88% of patients were seizure free. | Tsuji *et al*. 1993^31^ | Patients received preoperative prophylactic ASM. |
| Schneider *et al*. 2019^32^ | For preoperative seizures, 66% of patients had generalised and the remainder focal semiology. 90% were ILAE class one at follow up of mean 81 months. | Wang *et al*. 2018^33^ | 59% received prophylactic ASM intraoperatively which continued for 3 to 7 days. |
| Singh *et al*. 2023^34^ | 40% presented with focal aware seizures, and 60% had complex seizures or generalisation. 79% were *ILAE class 1 at last follow up.* | Wirsching *et al*. 2016^35^ | 46% of patients received prophylactic ASM. |
| Stevens *et al*. 1983^36^ | 43% had grand mal and 32% had focal preoperative seizures. | Yang *et al*. 2020^37^ | 37% of patients received prophylactic ASM 3 days before till 7 days after surgery. |
| Teske *et al*. 2024^29^ | 30% of all patients presented with focal or generalised seizures. | Zhang *et al*. 2020^38^ | Seizure naïve patients were given perioperative prophylactic ASM which continued for 2 weeks. |
| Wirsching *et al*. 2016^35^ | 63% of patients with early postoperative seizures suffered with late postoperative seizures meeting ILAE definitions for epilepsy. | Zhang *et al*. 2015^39^ | Patients received prophylactic ASM. |

### Online Resource 4: Results of meta-analyses with trim and fill (TF) correction and Egger’s tests

| **Analysis** | | **Sample**  ***k* (*n*)** | **Meta-analysis** | | | | | **Heterogeneity** | | | | **Egger’s test** | | |  |
| --- | --- | --- | --- | --- | --- | --- | --- | --- | --- | --- | --- | --- | --- | --- | --- |
|  |  |  | ***OR*** | **95% *CI*** | ***t*** | ***p*** | ***I*^2^ %** | | **τ^2^** | ***Q* (*p*)** | **Intercept (95% *CI)*** | | ***t*** | ***p*** |  |
| **Preoperative oedema and preoperative seizure** | Full | 32 (8,345) | 3.58 | 2.62-4.89 | 8.4 | <.001 | 67 | | 0.51 | 92.8 (<.001) | 0.51 (-0.81 - 1.84) | | 0.76 | .454 |  |
|  | Full TF | 40 | 2.93 | 2.12-4.04 | 6.8 | <.001 | 70 | | 0.71 | 129.4 (<.001) |  | |  |  |  |
|  | Excluding outliers^a^ | 28 (7,725) | 3.54 | 3.12-4.01 | 20.7 | <.001 | 0 | | 0.00 | 26.9 (.468) | 0.62 (-0.17 – 1.42) | | 1.54 | .136 |  |
|  | Excluding outliers^a^ TF | 35 | 3.36 | 2.94-3.84 | 18.4 | <.001 | 11 | | 0.02 | 38.3 (.282) |  | |  |  |  |
|  | Adjusted headache and gender^b,c^ | 3 (2,241) | 3.9 | 2.43-6.28 | 12.3 | .007 | 0 | | 0 | 0.8 (.662) | -1.54 (-4.11 - 1.03) | | 1.03 | 0.448 |  |
| **Preoperative oedema and all postoperative seizures** | Full | 32 (8,181) | 1.64 | 1.38-1.95 | 5.8 | <.001 | 65 | | 0.05 | 87.8 (<.001) | 1.27 (-0.87 – 1.67) | | 6.24 | <.001 |  |
|  | Full TF | 47 | 1.07 | 0.82-1.40 | 0.5 | .591 | 72 | | 0.51 | 162.1 (<.001) |  | |  |  |  |
|  | Excluding outliers^d^ | 31 (7,776) | 1.77 | 1.50-2.09 | 6.9 | <.001 | 10 | | 0.02 | 33.3 (.311) | 0.35 (0.56 – 1.24) | | 0.80 | .459 |  |
|  | Excluding outliers TF | 32 | 1.75 | 1.47-2.09 | 6.6 | <.001 | 14 | | 0.03 | 36.1 (.244) |  | |  |  |  |
| **Preop oedema early postop seizure** | Full | 9 (2,929) | 1.47 | 1.12-1.94 | 3.3 | .011 | 0 | | 0.00 | 4.5 (.813) | 0.48 (-0.74 – 1.68) | | 0.77 | .469 |  |
|  | Full TF | 11 | 1.35 | 1.03-1.78 | 2.4 | .036 | 0 | | 0.00 | 7 (.723) |  | |  |  |  |
| **Preop oedema late postop seizure** | Full | 9 (2,150) | 1.87 | 1.53-2.23 | 7.2 | <.001 | 0 | | 0.00 | 3.7 (.887) | 0.51 (-0.33 – 1.35) | | 1.19 | .272 |  |
|  | Full TF | 11 | 1.80 | 1.44-2.26 | 5.8 | <.001 | 0 | | 0.00 | 6.6 (.765) |  | |  |  |  |
| **Post-radiosurgery** | Full | 3 (376) | 10.93 | 0.57-211.25 | 3.5 | .074 | 42 | | 0.69 | 3.5 (.177) | 2.84 (-7.67 – 13.35) | | -0.53 | .690 |  |
| **Postoperative oedema** | Preoperative seizure^b^ | 4 (308) | 3.11 | 0.84-11.45 | 2.8 | .070 | 0 | | 0.00 | 2.0 (.577) | -0.5 (-3.69 – 2.70) | | -0.30 | .790 |  |
|  | Postoperative seizure^b^ | 3 (119) | 6.70 | 4.03-11.15 | 16.1 | .004 | 0 | | 0.00 | 0.1 (.954) | 0.03 (-1.15 – 1.20) | | 0.04 | .972 |  |
| ^a^ Outliers included Markovic *et al*.^40^, Stevens *et al.*^41^, Seyedi *et al.*^42^, Howng *et al.*^43^  ^b^ No additional studies from trim and fill correction  ^c^ Studies in this adjusted analysis had additional corrections which are presented in Online Resource 17  ^d^Outlier was Brokinkel *et al.* due to narrow confidence interval | | | | | | | | | | | | | | | |

### Online Resource 5: Predictors of seizure from studies with univariable analyses

| Proportion of studies (of k) with statistically significant positive (+ve), negative (-ve), or non significant (0), association with seizures at different time points | | | | | | | | | | | | | | | | | | | | | | | | | | | | | | | | | |
| --- | --- | --- | --- | --- | --- | --- | --- | --- | --- | --- | --- | --- | --- | --- | --- | --- | --- | --- | --- | --- | --- | --- | --- | --- | --- | --- | --- | --- | --- | --- | --- | --- | --- |
| Variables | | Preoperative seizure  (*k* = 22, *n* = 7,029) | | | | | | | | Early postoperative seizure  (*k* = 7, *n* = 2,813) | | | | | | | | Late postoperative seizure  (*k* = 5, *n* = 1,496) | | | | | | | | All postoperative seizure  (early late and unknown)  (*k* = 16, *n* = 5,960) | | | | | | | |
|  |  | *k* | | +ve | | 0 | | -ve | | *k* | | +ve | | 0 | | -ve | | *k* | | +ve | | 0 | | -ve | | *k* | | +ve | | 0 | | -ve | |
| Background | Age | 19 | 5% | | 79% | | 16% | | 5 | | 0% | | 80% | | 20% | | 5 | | 0% | | 60% | | 40% | | 12 | | 8% | | 75% | | 17% | |  |
|  | Male | 20 | 35% | | 60% | | 5% | | 6 | | 17% | | 83% | | 0% | | 5 | | 0% | | 100% | | 0% | | 14 | | 7% | | 93% | | 0% | |  |
|  | Comorbidity | 3 | 0% | | 67% | | 33% | | 2 | | 0% | | 100% | | 0% | |  | |  | |  | |  | | 2 | | 0% | | 100% | | 0% | |  |
|  | Performance status | 6 | 17% | | 50% | | 33% | | 4 | | 0% | | 75% | | 25% | |  | |  | |  | |  | | 6 | | 0% | | 67% | | 33% | |  |
|  | Multiple meningioma |  |  | |  | |  | |  | |  | |  | |  | | 1 | | 100% | | 0% | | 0% | | 2 | | 50% | | 50% | | 0% | |  |
| Symptoms | Headache | 6 | 0% | | 17% | | 83% | | 2 | | 0% | | 100% | | 0% | |  | |  | |  | |  | | 3 | | 0% | | 100% | | 0% | |  |
|  | Preoperative deficit | 7 | 0% | | 29% | | 71% | | 3 | | 33% | | 67% | | 0% | | 3 | | 67% | | 33% | | 0% | | 7 | | 43% | | 57% | | 0% | |  |
| Location | Convexity | 7 | 29% | | 71% | | 0% | | 3 | | 0% | | 100% | | 0% | | 4 | | 75% | | 25% | | 0% | | 8 | | 63% | | 38% | | 0% | |  |
|  | Falcine | 3 | 100% | | 0% | | 0% | |  | |  | |  | |  | | 1 | | 0% | | 100% | | 0% | | 3 | | 33% | | 67% | | 0% | |  |
|  | Sagittal | 5 | 60% | | 40% | | 0% | | 1 | | 0% | | 100% | | 0% | | 3 | | 33% | | 67% | | 0% | | 6 | | 33% | | 67% | | 0% | |  |
|  | Skull base | 9 | 0% | | 33% | | 67% | | 3 | | 0% | | 33% | | 67% | | 1 | | 0% | | 100% | | 0% | | 7 | | 0% | | 57% | | 43% | |  |
|  | Olfactory groove | 3 | 0% | | 100% | | 0% | |  | |  | |  | |  | |  | |  | |  | |  | |  | |  | |  | |  | |  |
|  | Suprasellar | 4 | 0% | | 50% | | 50% | |  | |  | |  | |  | |  | |  | |  | |  | |  | |  | |  | |  | |  |
|  | Sphenoid | 3 | 0% | | 100% | | 0% | |  | |  | |  | |  | |  | |  | |  | |  | | 1 | | 100% | | 0% | | 0% | |  |
|  | Clival | 2 | 0% | | 100% | | 0% | |  | |  | |  | |  | |  | |  | |  | |  | |  | |  | |  | |  | |  |
|  | Posterior fossa | 2 | 0% | | 0% | | 100% | |  | |  | |  | |  | | 1 | | 0% | | 0% | | 100% | | 2 | | 0% | | 50% | | 50% | |  |
|  | Ventricle | 3 | 0% | | 100% | | 0% | |  | |  | |  | |  | |  | |  | |  | |  | |  | |  | |  | |  | |  |
|  | Motor cortex |  |  | |  | |  | | 2 | | 100% | | 0% | | 0% | |  | |  | |  | |  | | 2 | | 100% | | 0% | | 0% | |  |
| Laterality | Left | 5 | 0% | | 100% | | 0% | |  | |  | |  | |  | | 1 | | 0% | | 100% | | 0% | | 4 | | 25% | | 75% | | 0% | |  |
|  | Right | 3 | 0% | | 100% | | 0% | |  | |  | |  | |  | | 1 | | 0% | | 100% | | 0% | | 2 | | 0% | | 100% | | 0% | |  |
|  | Midline/bilateral | 7 | 0% | | 71% | | 29% | | 2 | | 0% | | 100% | | 0% | | 1 | | 0% | | 100% | | 0% | | 4 | | 0% | | 100% | | 0% | |  |
| Tumour | Oedema | 21 | 95% | | 5% | | 0% | | 7 | | 14% | | 86% | | 0% | | 5 | | 20% | | 80% | | 0% | | 16 | | 44% | | 56% | | 0% | |  |
|  | Tumour size | 18 | 33% | | 67% | | 0% | | 6 | | 17% | | 83% | | 0% | | 4 | | 50% | | 50% | | 0% | | 13 | | 31% | | 69% | | 0% | |  |
|  | WHO grade | 11 | 36% | | 64% | | 0% | | 6 | | 17% | | 83% | | 0% | | 5 | | 60% | | 40% | | 0% | | 13 | | 46% | | 54% | | 0% | |  |
|  | Brain invasion | 5 | 60% | | 40% | | 0% | | 1 | | 0% | | 100% | | 0% | |  | |  | |  | |  | | 2 | | 50% | | 50% | | 0% | |  |
|  | Calcification | 3 | 33% | | 67% | | 0% | |  | |  | |  | |  | | 3 | | 0% | | 100% | | 0% | | 5 | | 0% | | 100% | | 0% | |  |
|  | Cystic | 2 | 50% | | 50% | | 0% | |  | |  | |  | |  | |  | |  | |  | |  | |  | |  | |  | |  | |  |
| Seizures | Preoperative seizures |  |  | |  | |  | | 5 | | 80% | | 20% | | 0% | | 4 | | 100% | | 0% | | 0% | | 13 | | 85% | | 15% | | 0% | |  |
|  | Early postop seizure |  |  | |  | |  | |  | |  | |  | |  | | 2 | | 0% | | 100% | | 0% | | 3 | | 0% | | 100% | | 0% | |  |
|  | Intraoperative seizure |  |  | |  | |  | |  | |  | |  | |  | |  | |  | |  | |  | | 1 | | 0% | | 100% | | 0% | |  |
| Anti seizure medication (ASM) | Preoperative |  |  | |  | |  | | 1 | | 100% | | 0% | | 0% | | 1 | | 100% | | 0% | | 0% | | 2 | | 100% | | 0% | | 0% | |  |
|  | Perioperative |  |  | |  | |  | | 3 | | 0% | | 100% | | 0% | |  | |  | |  | |  | | 2 | | 0% | | 100% | | 0% | |  |
|  | At discharge |  |  | |  | |  | |  | |  | |  | |  | | 3 | | 67% | | 0% | | 33% | | 3 | | 67% | | 0% | | 33% | |  |
| Prophylactic ASM | Perioperative |  |  | |  | |  | | 1 | | 100% | | 0% | | 0% | |  | |  | |  | |  | | 2 | | 50% | | 50% | | 0% | |  |
| Treatment | Simpson grade |  |  | |  | |  | | 6 | | 0% | | 100% | | 0% | | 5 | | 0% | | 80% | | 20% | | 15 | | 0% | | 93% | | 7% | |  |
|  | Postop deficit |  |  | |  | |  | | 4 | | 100% | | 0% | | 0% | | 2 | | 50% | | 50% | | 0% | | 6 | | 67% | | 33% | | 0% | |  |
|  | Radiotherapy |  |  | |  | |  | |  | |  | |  | |  | | 3 | | 67% | | 33% | | 0% | | 6 | | 50% | | 50% | | 0% | |  |
|  | Recurrence |  |  | |  | |  | |  | |  | |  | |  | | 5 | | 80% | | 20% | | 0% | | 9 | | 67% | | 33% | | 0% | |  |
|  | Complications |  |  | |  | |  | | 4 | | 75% | | 25% | | 0% | | 4 | | 50% | | 50% | | 0% | | 9 | | 56% | | 33% | | 11% | |  |
|  | Stroke |  |  | |  | |  | | 1 | | 100% | | 0% | | 0% | | 1 | | 0% | | 100% | | 0% | | 2 | | 50% | | 50% | | 0% | |  |
|  | Bleed |  |  | |  | |  | | 2 | | 100% | | 0% | | 0% | | 2 | | 0% | | 100% | | 0% | | 6 | | 50% | | 50% | | 0% | |  |
|  | Infection |  |  | |  | |  | |  | |  | |  | |  | | 1 | | 100% | | 0% | | 0% | | 3 | | 33% | | 67% | | 0% | |  |
|  | Hydrocephalus |  |  | |  | |  | | 1 | | 100% | | 0% | | 0% | | 2 | | 50% | | 50% | | 0% | | 5 | | 60% | | 40% | | 0% | |  |
|  | Recraniotomy |  |  | |  | |  | |  | |  | |  | |  | |  | |  | |  | |  | | 1 | | 100% | | 0% | | 0% | |  |
| Preoperative seizure studies  Baumgarten^44^ (*n =* 420)  Bogdanovic^45^ (*n =* 333)  Chaichana^46^ (*n =* 626)  Chen^47^ (*n =* 843)  Gupte^48^ (*n =* 356)  Hamasaki^49^ (*n =* 100)  Hinrichs^50^ (*n =* 499)  Hwang & Joo^51^ (*n =* 303)  Islim^52^ (*n =* 283)  Kawaguchi^53^ (*n =* 61)  Kemerdere^54^ (*n =* 63) | | Li & Wang^55^ (*n =* 772)  Lieu^56^ (*n =* 214)  Morsy^57^ (*n =* 40)  Nassar^58^ non Skull base(*n =* 154) and skull base (*n =* 90)  Pauletto^59^ (*n =* 358)  Seyedi^42^ (*n =* 295)  Teske^60^ (*n =* 95)  Wach^61^ (*n =* 330)  Wang^62^ (*n =* 102)  Wirsching^63^ (*n =* 692) | | | | | | | | Early postoperative  Abzalova^64^ (*n =* 56)  Bogdanovic^45^ (*n =* 333)  Cai^65^ (*n =* 517)  Chen^47^ (*n =* 773)  Li & Wang^55^ (*n =* 772)  Wang^62^ (*n =* 102)  Xu^66^ (*n =* 260) | | | | | | | | Late postoperative  Bogdanovic^45^ (*n =* 333)  Hwang & Joo^51^ (*n =* 303)  Wang^62^ (*n =* 102)  Wirsching^63^ (*n =* 773)  Zhang & Li^67^ (*n =* 318) | | | | | | | | All postoperative  Early (minus Bogdanovic^45^ and Wang^62^), late and unknown  Unknown:  Baumgarten^44^ (*n =* 420)  Gupte^48^ (*n =* 356)  Islim^52^ (*n =* 283)  Lieu^56^ (*n =* 213)  Seyedi^42^ (*n =* 295)  Yang^68^ (*n =* 186) | | | | | | | |

### Online Resource 6: Predictors of seizure from studies with multivariable analyses

| Proportion of studies (of k) with statistically significant positive (+ve), negative (-ve), or non significant (0), association with seizures at different time points | | | | | | | | | | | | | | | | | |
| --- | --- | --- | --- | --- | --- | --- | --- | --- | --- | --- | --- | --- | --- | --- | --- | --- | --- |
| Variables | | Preoperative seizure  (*k* = 16, *n* = 6,373) | | | | Early postoperative seizure  (*k* = 5, *n* = 2,604) | | | | Late postoperative seizure  (*k* = 6, *n* = 1,892) | | | | All postoperative seizure  (early late and unknown)  (*k* = 15, *n* = 5,703) | | | |
|  |  | *k* | +ve | 0 | -ve | *k* | +ve | 0 | -ve | *k* | +ve | 0 | -ve | *k* | +ve | 0 | -ve |
| Background | Age | 10 | 30% | 40% | 30% | 2 | 0% | 50% | 50% | 2 | 0% | 50% | 50% | 5 | 0% | 80% | 20% |
|  | Male | 12 | 8% | 92% | 0% | 2 | 0% | 100% | 0% | 2 | 0% | 100% | 0% | 4 | 0% | 100% | 0% |
|  | Performance status | 3 | 0% | 33% | 67% |  |  |  |  |  |  |  |  | 1 | 0% | 100% | 0% |
|  | Headache | 6 | 0% | 0% | 100% |  |  |  |  |  |  |  |  |  |  |  |  |
|  | Preoperative deficit | 8 | 0% | 50% | 50% |  |  |  |  |  |  |  |  |  |  |  |  |
| Location | Convexity | 3 | 0% | 100% | 0% | 1 | 0% | 100% | 0% | 3 | 33% | 67% | 0% | 6 | 50% | 50% | 0% |
|  | Falcine | 4 | 25% | 75% | 0% |  |  |  |  |  |  |  |  | 1 | 100% | 0% | 0% |
|  | Sagittal | 4 | 25% | 75% | 0% |  |  |  |  |  |  |  |  |  |  |  |  |
|  | Skull base | 6 | 0% | 50% | 50% | 2 | 0% | 50% | 50% |  |  |  |  | 4 | 25% | 25% | 50% |
|  | Suprasellar | 3 | 0% | 67% | 33% |  |  |  |  |  |  |  |  |  |  |  |  |
|  | Sphenoid wing |  |  |  |  |  |  |  |  |  |  |  |  | 1 | 100% | 0% | 0% |
|  | Motor cortex | 1 | 100% | 0% | 0% | 2 | 100% | 0% | 0% |  |  |  |  | 2 | 100% | 0% | 0% |
|  | Left | 1 | 0% | 100% | 0% |  |  |  |  | 1 | 0% | 100% | 0% | 2 | 50% | 50% | 0% |
|  | Midline | 2 | 0% | 100% | 0% |  |  |  |  |  |  |  |  |  |  |  |  |
| Tumour | Oedema | 16 | 81% | 19% | 0% | 3 | 33% | 67% | 0% | 3 | 33% | 67% | 0% | 9 | 22% | 78% | 0% |
|  | Tumour size | 9 | 22% | 67% | 11% | 3 | 33% | 67% | 0% | 2 | 100% | 0% | 0% | 7 | 57% | 43% | 0% |
|  | WHO grade | 4 | 0% | 100% | 0% | 1 | 0% | 100% | 0% | 4 | 0% | 100% | 0% | 5 | 0% | 100% | 0% |
|  | Brain invasion |  |  |  |  |  |  |  |  |  |  |  |  | 1 | 0% | 100% | 0% |
| Seizure | Preoperative |  |  |  |  | 2 | 50% | 50% | 0% | 6 | 66% | 33% | 0% | 12 | 58% | 42% | 0% |
|  | Early postoperative |  |  |  |  |  |  |  |  | 1 | 100% | 0% | 0% | 1 | 100% | 0% | 0% |
| Antiseizure medication (ASM) | Preoperative |  |  |  |  | 1 | 0% | 100% | 0% | 2 | 0% | 100% | 0% | 2 | 0% | 100% | 0% |
|  | After discharge |  |  |  |  |  |  |  |  | 3 | 33% | 33% | 33% | 3 | 33% | 33% | 33% |
| Prophylactic ASM | Perioperative |  |  |  |  | 1 | 0% | 0% | 100% |  |  |  |  | 2 | 50% | 0% | 50% |
| Treatment | Simpson grade |  |  |  |  | 1 | 0% | 100% | 0% | 3 | 0% | 100% | 0% | 3 | 0% | 100% | 0% |
|  | Radiotherapy |  |  |  |  |  |  |  |  | 2 | 0% | 100% | 0% | 4 | 25% | 75% | 0% |
|  | Recurrence |  |  |  |  |  |  |  |  | 5 | 60% | 40% | 0% | 7 | 57% | 43% | 0% |
|  | Complications |  |  |  |  | 3 | 100% | 0% | 0% | 4 | 50% | 50% | 0% | 8 | 75% | 25% | 0% |
|  | Bleed |  |  |  |  |  |  |  |  | 1 | 100% | 0% | 0% | 1 | 100% | 0% | 0% |
|  | Recraniotomy |  |  |  |  |  |  |  |  | 2 | 50% | 50% | 0% | 3 | 33% | 67% | 0% |
|  | Infection |  |  |  |  |  |  |  |  | 1 | 100% | 0% | 0% | 1 | 100% | 0% | 0% |
|  | Hydrocephalus |  |  |  |  |  |  |  |  | 1 | 100% | 0% | 0% | 2 | 100% | 0% | 0% |
|  | Postoperative KPS |  |  |  |  | 1 | 0% | 0% | 100% |  |  |  |  | 1 | 0% | 0% | 100% |
|  | Postoperative deficit |  |  |  |  | 1 | 0% | 100% | 0% | 1 | 100% | 0% | 0% | 3 | 33% | 67% | 0% |
| Preoperative seizure studies  Ahmeti^69^ (*n =* 696)  Baumgarten^44^ (*n =* 420)  Bogdanovic^45^ (*n =* 333)  Chaichana^46^ (*n =* 626)  Chen^47^ (*n =* 843)  Gupte^48^ (*n =* 356)  Hamasaki^49^ (*n =* 100)  Hinrichs^50^ (*n =* 499) | | Hwang & Joo^51^ (*n =* 303)  Islim^52^ (*n =* 283)  Kemerdere^54^ (*n =* 63)  Li & Wang^55^ (*n =* 772)  Pauletto^59^ (*n =* 358)  Seyedi^42^ (*n =* 296)  Teske^60^ (*n =* 95)  Wach^61^ (*n =* 330) | | | | Early postoperative  Bogdanovic^45^ (*n =* 333)  Cai^65^ (*n =* 517)  Chen^47^ (*n =* 773)  Li & Wang^55^ (*n =* 772)  Zhang & Wang^70^ (*n =* 209) | | | | Late postoperative  Bogdanovic^45^ (*n =* 333)  Hwang & Joo^51^ (*n =* 303)  Kemerdere^54^ (*n =* 57)  Wang^62^ (*n =* 102)  Wirsching^63^ (*n =* 779)  Zhang & Li^71^ (*n =* 318) | | | | All postoperative  Early (minus Bogdanovic^45^), late and unknown  Unknown  Baumgarten^44^ (*n =* 420)  Gupte^48^ (*n =* 356)  Islim^52^ (*n =* 283)  Seyedi^42^ (*n =* 295)  Yang^68^ (*n =* 186) | | | |

### Online Resource 7: Findings from narrative review

| **Seizure** | **Author** | **Findings in narrative review** |
| --- | --- | --- |
| Preop | Ahmed *et al*. 2023^72^ | No association between preop oedema and preop seizure |
|  | Goertz *et al*. 2018^73^ | Positive and significant association between preop oedema and preop seizure in univariable and multivariable tests. |
|  | Lobato *et al*. 1996^74^ | Positive and significant association between preop oedema and preop seizure in univariable and multivariable tests. |
|  | Loewenstern *et al*. 2019^75^ | No association between preop oedema and preop seizure |
|  | Salpietro *et al*. 1997^76^ | "Finger like" oedema preop associated with preop seizures in univariable testing |
|  | Simis *et al*. 2008^77^ | No association between preop oedema and preop seizure |
| Eps | Ersoy *et al*. 2020^78^ | No significant association between preop oedema and EPS. 25% of those with EPS had further seizures in a year of which 20% had oedema. |
|  | Lazzarin *et al*. 2022^79^ | Positive significant association between preop oedema and EPS seizure |
| Aps | Asemota *et al*. 2022^80^ | Preop oedema is a significant predictor of readmissions due to seizures within 30 days of surgery |
|  | Goertz *et al*. 2023^81^ | Three patients had postop seizure, none had oedema preop. |
|  | Hess *et al*. 2019^82^ | Preop oedema volume not associated with postop seizure frequency. |
|  | Le *et al*. 2023^83^ | Preop oedema predictor of postop seizure. |
|  | Xue *et al*. 2018^84^ | Preop oedema significant predictor or postop seizure in univariable but not multivariable regression. |
| Unclear | Kirn *et al*. 1998^85^ | No significant association between oedema and seizures (unclear if pre or postop) |
|  | Skardelly *et al*. 2017^86^ | No significant association between oedema and seizures (unclear if pre or postop) |
| SRS | Conti *et al*. 2016^87^ | Post-SRS oedema occurred in 8%, 89% of which developed seizure. |
|  | Jung *et al*. 2022^88^ | Post-SRS oedema occurred in 8%, 20% of which developed seizure. |
|  | Patil *et al*. 2008^89^ | Post-SRS oedema occurred in 15%, 27% of which developed seizure. |
|  | Zachenhofer *et al*. 2006^90^ | None developed post-SRS oedema, one patient had a seizure three years later. |
| Pre = Preoperative, EPS = early postoperative, APS = any postoperative, SRS = radiosurgery, Unclear = unclear if pre or postop | | |

### Online Resource 8: Study selection for all postoperative seizures

| Author | Postoperative seizure timing | Seizure follow up (for late/unknown) | | Preoperative Seizure | |
| --- | --- | --- | --- | --- | --- |
|  |  | (months) | Range (months) |  |  |
| Abzalova *et al.* 2023^64^ | Early |  |  | Absent |  |
| Ahmeti *et al.* 2023^69^ | Unknown | 24 (median) | ?-203 |  | |
| Baumgarten *et al.* 2021^44^ | Unknown | 21 (median) | 0-286 |  | |
| Bogdanovic *et al.* 2023^45^ | Late (Early excluded) | 78 (median) | (SD 43) |  | |
| Brokinkel *et al.* 2021^91^ | Unknown | 9 (median) | 2-29 | Absent | |
| Cai *et al.* 2022^65^ | Early |  |  | Absent | |
| Chen *et al.* 2017^47^ | Early (Unknown excluded) |  |  |  | |
| Frati *et al.* 2022^92^ | Unknown | 46 (unclear) |  |  | |
| Gadot *et al.* 2021^93^ | Late | 17 (median) | 3-30 | Present | |
| Güngör *et al.* 2019^94^ | Early |  |  |  | |
| Gupte *et al.* 2021^48^ | Unknown | 21 (median) | 15-27 |  | |
| Hwang & Joo *et al.* 2019^51^ | Late | 49 (mean) | 1-137 |  | |
| Islim *et al.* 2018^52^ | Unknown |  | 12-? | Split present and absent | |
| Li & Wang *et al.* 2020^55^ | Early (Unknown excluded) |  |  |  | |
| Lieu *et al.* 2000^56^ | Unknown |  | 1 - 12 |  | |
| McKevitt *et al.* 2023^95^ | Unknown | 31 (mean) | 27 (SD) | Absent | |
| Nassar *et al.* 2022^96^ | Late | At 3 months |  | Split present and absent | |
| Rajab *et al.* 2022^97^ | Unknown |  |  |  | |
| Schneider *et al.* 2019^98^ | Late | At 12 months |  | Present | |
| Seyedi *et al.* 2018^42^ | Unknown |  |  |  | |
| Singh *et al.* 2023^99^ | Unknown |  |  | Present | |
| Teske *et al.* 2024^60^ | Late (Early excluded) | 21 (median) | 1-128 |  | |
| Tsuji *et al.* 1993^100^ | Unknown |  |  |  | |
| Wang *et al.* 2018^62^ | Late (Early excluded) | 78 (mean) | 5-195 |  | |
| Wirsching *et al.* 2016^63^ | Late | 67 (median) | (CI 63-72) | Split present and absent | |
| Xu *et al.* 2021^66^ | Early |  |  |  | |
| Yang *et al.* 2020^68^ | Unknown |  | 0-14 days | Split present and absent | |
| Zhang & Li *et al.* 2020^67^ | Late | 27 (median) | 6-56 |  | |
| *Blank cells = unspecified* | | | | | |

### Online Resource 9: Reports with radiotherapy

| Study | Narrative review | Covariate review | Meta-analysis | Treatment | Prior surgery (%) | Tumour location | Treatment plan | Prescription dose in gy median (range) | Isodose median % (range) | Maximum dose  median (range) | Standard post-treatment imaging | % Oedema | % Seizure | % Seizure + oedema |  |
| --- | --- | --- | --- | --- | --- | --- | --- | --- | --- | --- | --- | --- | --- | --- | --- |
| Conti *et al*.  2016^87^ (*n* = 229) | 1 |  |  | Cyberknife | 68 |  | single, multiple, hypofractionated^a^ | 27 (14-48) | 78 (62-86) | 28 (15-64) | 3m, 6m for 2y | 8 |  | 7 |  |
| Jung *et al*.  2022^88^ (*n* = 127) | 1 |  |  | Gamma knife |  | Only convexity | single | 14 | 50 |  | at 6 and 12 months | 8 |  | 2 |  |
| Patil *et al*.  2008^89^(*n* = 102) | 1 |  |  | Cyberknife | 45 | No infratentorial | usually 2-5 sessions | 18 (11-25) | index 1.5 | 22 (14-39) | 6m for 1y then yearly | 15 |  | 4 |  |
| Zachenhofer *et al*. 2006^90^ (*n* = 43) | 1 |  |  | Gamma knife | 69 | Skull base only | single | 17 (9-25) | 50 (40-65) | 34 (20-50) | 6m for 1y then yearly | 0 | 2 | 0 |  |
| Kollova *et al*.  2007^101^ (*n* = 368) |  | 1 |  | Gamma knife | 30 | Some infratentorial | single (larger had staged) | 13 (7-24) | 50 | 24 (13-45) | 6m for 1y then yearly | 15 | 2 |  |  |
| Ding *et al*.  2013^102^ (*n* = 49) |  |  | 1 | Gamma knife | 80 | Parasagittal parafalcine | Single (51%) | 15 (10 -20) | 40 (30-35) |  | 6m for 2y then yearly | 20 | 14 | 6 |  |
| Hwang & Kim *et al*. 2019^103^ (*n* = 133) |  | 1 | 1 | Gamma knife | 16 | No infratentorial |  | 14 (14 -20) | 50 |  | 1,3,6,12 m then yearly | 32 | 8 | 5 |  |
| Kuhn *et al*.  2014^104^ (*n* = 194) |  |  | 1 | Gamma knife | 41 | Some infratentorial | single (some had multiple) | 12 (9-20) |  |  | 6m then yearly | 16 | 5 | 3 |  |
| ^a^Some patients had hypofractionated stereotactic radiotherapy | | | | | | | | | | | | | | | |

### Online Resource 10: Subgroup analysis preoperative seizures

| **Preoperative seizure and preoperative oedema** | | | | | | | **Heterogeneity tests** | | |
| --- | --- | --- | --- | --- | --- | --- | --- | --- | --- |
|  | | **Sample**  ***k* (*n*)** | ***OR* (95% CI)** | ***t* (*df*) *p*** | **Between group^a^** | **Prediction interval, 95%** | ***I*^2^*%***  ***(95%CI)*** | **τ^2^** | ***Q (df) p*** |
| Risk of bias | Low | 4 (1,881) | 2.55 (1.76-3.69) | 8.1 (3) .004 | *Q*=31.1  *df*=3  *p*<.001 | 1.44-4.52 | 0 (0-85) | 0.00 | 2.3 (3) .514 |
|  | Some | 10 (3,445) | 4.23 (3.71-4.82) | 25.0 (9) <.001 |  | 3.41-5.26 | 0 (0-62) | 0.00 | 3.4 (9) .946 |
|  | High | 12 (2,340) | 3.41 (2.75-4.22) | 12.5 (11) <.001 |  | 2.71-4.28 | 0 (0-58) | 0.00 | 10.0 (11) .533 |
|  | Very high | 2 (59) | 7.58 (0.79-73.20) | 11.3 (1) .056 |  | - | 0 (-) | 0.00 | <0.1 (1) .783 |
| Continent | Africa | 1 (40) | 7.00 (1.74-28.17) | 2.7 (-) .006 | *Q*=1.40  *df*=3  *p*=.706 | - | - | - | - |
|  | Asia | 8 (1,391) | 3.46 (2.89-4.14) | 16.3 (7) <.001 |  | 2.42-4.95 | 0 (0-68) | 0.00 | 1.9 (7) .965 |
|  | Europe | 16 (4,469) | 3.67 (2.98-4.52) | 13.3 (15) <.001 |  | 2.30-5.84 | 25 (0-59) | 0.04 | 20.1 (11) .169 |
|  | N. America | 3 (1,825) | 3.23 (1.48-7.03) | 6.5 (2) .023 |  | 0.10-97.02 | 47 (0-84) | 0.04 | 3.7 (2) .154 |
| Infratentorial allowed | Yes | 14 (4,275) | 3.26 (2.78-3.82) | 16.1 (13) <.001 | *Q*=2.72  *p*=.100 | 2.73-3.88 | 0 (0-55) | 0.00 | 10.8 (13) .629 |
|  | No | 14 (3,450) | 3.98 (3.23-4.91) | 14.2 (13) <.001 |  | 3.08-5.15 | 5 (0-57) | 0.00 | 13.6 (13) .401 |
| Oedema scan | MRI | 17 (6,244) | 3.52 (2.95-4.20) | 15.1 (16) <.001 | *Q=0.37*  *df=2*  *p=.830* | 2.41-5.13 | 22 (0-56) | 0.03 | 20.5 (16) .197 |
|  | MRI or CT | 5 (972) | 3.81 (2.42-5.99) | 8.2 (4) .001 |  | 2.18-6.64 | 3 (0-80) | 0.00 | 4.1 (4) .388 |
|  | CT | 4 (374) | 3.27 (1.78-6.01) | 6.2 (3) .008 |  | 1.19-8.98 | 0 (0-85) | 0.00 | 2.0 (3) .576 |
|  | Unclear | 2 (135) | 3.60 (2.93-4.41) | 79.7 (1) .008 | - | - | 0 (-) | 0.00 | 0.0 (1) .966 |
| Oedema measure | Binary | 18 (4,361) | 3.30 (2.81-3.88) | 15.6 (17) <.001 | *Q*=4.03  *df*=2  *p*=133 | 2.77-3.93 | 0 (0-50) | 0.00 | 14.7 (17) .616 |
|  | Threshold | 8 (2,804) | 3.79 (2.7-5.32) | 8.2 (7) <.001 |  | 1.91-7.50 | 31 (0-69) | 0.05 | 10.2 (7) .180 |
|  | Area/volume | 2 (560) | 4.19 (1.31-13.38) | 15.7 (1) .041 |  | - | 0 (-) | 0.00 | 0.2 (1) .634 |
| Seizure described | Yes | 9 (2,725) | 3.64 (2.74-4.84) | 10.5 (8) <.001 | *Q*=0.04  *p*=.849 | 2.24-5.93 | 20 (0-62) | 0.03 | 10.0 (8) .262 |
|  | No | 19 (5,000) | 3.54 (3.03-4.14) | 17.1 (18) <.001 |  | 3.02-4.16 | 0 (0-49) | 0.00 | 16.9 (18) .531 |
| ^a^Result for subgroup differences excludes studies that are “Unclear” | | | | | | | | | |

### Online Resource 11: Subgroup analysis postoperative seizures

| Preoperative oedema and postoperative seizure | | **Sample**  ***k* (*n*)** | ***OR* (95% CI)** | ***t* (*df*) *p*** | **Prediction interval, 95%** | **Between group difference^a^** | **Heterogeneity tests** | | |  |
| --- | --- | --- | --- | --- | --- | --- | --- | --- | --- | --- |
|  |  |  |  |  |  |  | ***I*^2^*%* (95%*CI*)** | **τ^2^** | ***Q (df) p*** |  |
| Preoperative  seizure | Present | 7 (911) | 1.84 (0.81-4.18) | 1.8 (6) .120 | 0.32-10.67 | *Q* = 0.01  *p* = .932 | 53 (0-80) | 0.36 | 12.9 (6) .045 |  |
|  | Absent | 7 (1,576 | 1.90 (1.35-2.66) | 4.6 (6) .004 | 1.24-2.89 |  | 0 (0-71) | 0.00 | 4.4 (6) .630 |  |
|  | Unclear | 17 (5,289) | 1.75 (1.42-2.16) | 5.7 (16) <.001 | 1.42-2.16 | - | 0 (0-51) | 0.00 | 15.7 (16) .473 |  |
| Postoperative  seizure | Early | 6 (2,399) | 1.36 (0.95-1.97) | 2.2 (5) .081 | 0.83-2.24 | *Q* = 0.88  *p* = .348 | 0 (0-75) | 0.00 | 3.2 (5) .672 |  |
|  | Late | 11 (2,150) | 1.63 (1.25-2.12) | 4.1 (10) .002 | 1.2-2.19 |  | 0 (0-60) | 0.00 | 8.1 (10) .622 |  |
|  | Unclear | 14 (3,227) | 2.07 (1.54-2.78) | 5.3 (13) <.001 | 1.1-3.87 | - | 26 (0-61) | 0.06 | 17.7 (13) .170 |  |
| Infratentorial  tumour | Allowed | 13 (3,426) | 1.73 (1.32-2.28) | 4.4 (12) <.001 | 1.02-2.95 | *Q* = 0.01  *p* = .912 | 21 (0-59) | 0.04 | 15.2 (12) .230 |  |
|  | Excluded | 16 (3,757) | 1.77 (1.33-2.35) | 4.3 (15) <.001 | 1.14-2.73 |  | 12 (0-49) | 0.02 | 17.0 (15) .321 |  |
|  | Unclear | 2 (593) | 2.09 (0.24-17.93) | 4.4 (1) .143 | - | - | 0 (-) | 0.00 | 0.5 (1) .481 |  |
| Continent | Asia | 12 (3,120) | 1.91 (1.50-2.44) | 5.9 (11) <.001 | 1.47-2.49 | *Q* =1.0  *df* =2  *p* .597 | 0 (0-58) | 0.00 | 9.5 (11) .578 |  |
|  | Europe | 15 (3,357) | 1.63 (1.22-2.17) | 3.6 (14) .003 | 0.91-2.90 |  | 25 (0-59) | 0.05 | 18.6 (14) .182 |  |
|  | N. America | 4 (1,299) | 2.05 (0.78-5.41) | 2.3 (3) .100 | 0.34-12.27 |  | 29 (0-74) | 0.08 | 4.2 (3) .239 |  |
| Scan | MRI | 21 (6,130) | 1.78 (1.43-2.22) | 5.5 (20) <.001 | 1.18-2.68 | *Q* = 3.6  *p* = .058 | 14 (0-48) | 0.03 | 23.1 (20) .282 |  |
|  | CT | 2 (232) | 2.69 (0.24-30.12) | 5.2 (1) .121 | - |  | 0 (-) | 0.00 | 0.4 (1) .552 |  |
|  | MRI or CT | 6 (999) | 1.56 (0.90-2.72) | 2.1 (5) .092 | 0.59-4.14 | - | 30 (0-72) | 0.08 | 7.2 (5) .209 |  |
|  | Unclear | 2 (415) | 1.88 (0.73-4.83) | 8.5 (1) .080 | - |  | 0 (-) | 0.00 | 0.1 (1) .787 |  |
| Oedema measurement | Binary | 17 (3,640) | 1.86 (1.49-2.33) | 5.9 (16) <.001 | 1.38-2.51 | *Q* =0.4  *df* =2  *p* .810 | 6 (0-41) | 0.01 | 17.1 (16) .378 |  |
|  | Threshold | 11 (3,872) | 1.65 (1.18-2.31) | 3.3 (10) .008 | 0.80-3.39 |  | 28 (0-64) | 0.08 | 13.8 (10) .182 |  |
|  | Volume | 3 (264) | 1.78 (0.29-10.78) | 1.4 (2) .304 | 0.01-525.39 |  | 0 (0-90) | 0.00 | 1.8 (2) .416 |  |
| Prophylactic  ASM | Permitted | 15 (4,596) | 1.69 (1.38-2.07) | 5.5 (14) <.001 | - | *Q* = 0.2  *p* = .656 | 0 (0-54) | 0.00 | 10.8 (14) .704 |  |
|  | Excluded | 4 (1,026) | 1.91 (0.83-4.42) | 2.5 (3) .091 | 1.09-3.84 |  | 50 (0-83) | 0.10 | 6.0 (3) .112 |  |
|  | Unclear | 5 (1,243) | 2.05 (1.31-3.20) | 4.5 (4) .011 | 1.34-2.13 | - | 0 (0-79) | 0.00 | 2.7 (4) .619 |  |
|  | NA^b^ | 7 (911) | 1.84 (0.81-4.18) | 1.8 (6) .120 | 0.32-11.4 | - | 53 (0-80) | 0.36 | 12.9 (6) .045 |  |
| Bias | Low | 8 (2,632) | 1.75 (1.18-2.60) | 3.4 (7) .012 | 0.92-3.34 | *Q* =2.2  *df* =3  *p* .531 | 27 (0-67) | 0.04 | 9.6 (7) .212 |  |
|  | Some | 11 (3,188) | 1.59 (1.12-2.28) | 2.9 (10) .015 | 0.77-3.30 |  | 25 (0-63) | 0.08 | 13.3 (10) .208 |  |
|  | High | 9 (1,286) | 2.07 (1.40-3.05) | 4.3 (8) .003 | 1.37-3.11 |  | 0 (0-65) | 0.00 | 7.6 (8) .478 |  |
|  | Very high | 3 (670) | 2.05 (1.30-3.24) | 6.8 (2) .021 | 0.14-29.85 |  | 0 (0-90) | 0.00 | 0.5 (2) .776 |  |
| ^a^ Subgroup differences exclude studies with “Unclear”, ”NA”, ”MRI or CT” variables and *df* = 1 unless stated otherwise  ^b^ Subjects with preoperative seizures received anti-seizure treatment instead of prophylaxis | | | | | | | | | | |

### Online Resource 12: Univariable meta-regression for preoperative oedema and preoperative seizure

| Univariable  *k* = 28, *n* = 7,725 | | | Model results | | *Variance* | | | | | |
| --- | --- | --- | --- | --- | --- | --- | --- | --- | --- | --- |
| Study characteristic | | | *β* (*SE*) | *t* (*p*)  *df = 26* | $R_{*}^{2}$ | *I^2^* | τ^2^ | *H^2^* | *QE* (*p*)  *df = 26* |  |
| Infratentorial tumours | | Intercept | 1.38 (0.09) | 15.1 (<.001) | 0% | 0% | 0.00 | 1.00 | 24.4 (.553) |  |
|  |  | Allowed (*k* = 14, *n* =4,275) | -0.20 (0.12) | -1.6 (.113) |  |  |  |  |  |  |
| MRI to measure oedema | Complete^a^ case analysis | Intercept | 1.19 (0.25) | 4.8 (<.001) | 0% | 15% | 0.02 | 1.17 | 22.5 (.259) |  |
|  |  | Only MRI (*k* = 17, *n* = 6,244) | 0.06 (0.26) | 0.2 (.807) |  |  |  |  |  |  |
|  | Multiple imputation^b^ | Intercept | 1.33 (0.19) | 7.2 (<.001) | 0% | 2% | 0.00 | 1.02 | 26.6 (.426) |  |
|  |  | Only MRI (*k* = 19.8) | -0.08 (0.20) | -0.4 (.714) |  |  |  |  |  |  |
| Binary oedema measurement | | Intercept | 1.35 (0.09) | 14.9 (<.001) | 0% | 0% | 0.00 | 1.00 | 25.3 (.501) |  |
|  |  | Included (*k* = 18, *n* = 4,361) | -0.16 (0.12) | -1.3 (.210) |  |  |  |  |  |  |
| Use of seizure description or definition | | Intercept | 1.26 (0.08) | 16.1 (<.001) | 0% | 3% | 0.00 | 1.03 | 26.9 (.413) |  |
|  |  | Included (*k* = 9, *n* = 2,725) | 0.00 (0.13) | 0.0 (.988) |  |  |  |  |  |  |
| Risk of bias | | Intercept | 1.28 (0.08) | 16.2 (<.001) | 0% | 3% | 0.00 | 1.03 | 26.9 (.416) |  |
|  |  | High/very high (*k* = 14, n = 2,399) | -0.03 (0.13) | -0.2 (.836) |  |  |  |  |  |  |
| ^a^7 studies omitted, therefore total *K* = 21, total *n* = 6,618, *df* = 19  *^b^k* for multiple imputation is taken from complete case series and average number of imputed studies. Total *k* is 28.1. | | | | | | | | | | |

### Online Resource 13: Multivariable meta-regression for preoperative oedema and preoperative seizure

| Forced entry multivariable analysis | Complete case analysis | | Multiple imputation | |
| --- | --- | --- | --- | --- |
|  | *K* = 21, *n* = 6,618 | | *k* = 28.1 | |
| $R_{*}^{2}$ | 0% | | 0% | |
| *F* (*p)*  *df*  *Coefficients* | 0.43 (.822)  *df*1 = 5, *df*2 = 15  2:6 | | 0.69 (.639)  *df*1 = 5, *df*2 = 22  2:6 | |
| *I^2^* | 22% | | 4% | |
| τ^2^ | 0.03 | | 0.01 | |
| *H^2^* | 1.28 | | 1.05 | |
| *QE*(*df*)p | 20.0 (15) .174 | | 23.3 (22) .498 | |
| Study characteristic | *β (SE)* | *t* (*p*) | *β (SE)* | *t* (*p*) |
| Intercept | 1.37 (0.33) | 4.1 (<.001) | 1.47 (0.24) | 6.2 (<.001) |
| Infratentorial tumours allowed | -0.14 (0.17) | -0.8 (.434) | -0.18 (0.14) | -1.3 (.212) |
| MRI to measure oedema | 0.02 (0.30) | 0.1 (.938) | -0.06 (0.23) | -0.3 (.794) |
| Binary oedema measurement | -0.12 (0.17) | -0.7 (.504) | -0.11 (0.14) | -0.8 (.409) |
| Seizure definition/description | 0.10 (0.18) | 0.5 (.613) | 0.04 (0.15) | 0.3 (.765) |
| High or very high risk of bias | -0.11 (0.21) | -0.5 (.621) | 0.00 (0.16) | 0.0 (.985) |

### Online Resource 14: Univariable meta-regression for preoperative oedema and postoperative seizures

| Univariable | | | Model results | | *Variance* (PM) | | | | |
| --- | --- | --- | --- | --- | --- | --- | --- | --- | --- |
| Study characteristic  *k* (*n*)  *df* = *k* - 2 | | Variable  *k* (*n*) | *β* (*SE*) | *t* (*p*) | $R_{*}^{2}$ | *Residual* | | | |
|  |  |  |  |  |  | *I^2^* | τ^2^ | *H^2^* | *QE* (*p*) |
| Postoperative  Seizure | Complete case  17 (4,547) | Intercept | 0.31 (0.20) | 2.0 (.063) | 0% | 0% | 0.00 | 1.00 | 11.3 (.734) |
|  |  | Late: 11 (2,150) | 0.17 (0.20) | 0.9 (.380) |  |  |  |  |  |
|  | Multiple imputation  *k* = 31 | Intercept | 0.49 (0.2) | 2.8 (.010) | 11% | 9% | 0.02 | 1.1 | 32.3 (.288) |
|  |  | Late (*k* = 21.1) | 0.12 (0.2) | 0.6 (.578) |  |  |  |  |  |
| Preoperative  Seizure | Complete case  14 (2,487) | Intercept | 0.64 (0.2) | 2.9 (.014) | 0% | 31% | 0.11 | 1.44 | 17.2 (.142) |
|  |  | Present: 7 (911) | -0.10 (0.3) | -0.3 (.773) |  |  |  |  |  |
|  | Multiple imputation  *k* = 31 | Intercept | 0.58 (0.1) | 4.6 (<.001) | 3% | 10% | 0.02 | 1.11 | 32.8 (.278) |
|  |  | Present (*k* = 15.5) | -0.02 (0.2) | -0.1 (.917) |  |  |  |  |  |
| Infratentorial  Tumours | Complete case  29 (7,183) | Intercept | 0.57 (0.1) | 4.2 (<.001) | 0% | 14% | 0.03 | 1.16 | 32.2 (.225) |
|  |  | Allowed: 13 (3,426) | -0.03 (0.2) | -0.1 (.892) |  |  |  |  |  |
|  | Multiple imputation  *k* = 31 | Intercept | 0.58 (0.1) | 4.5 (<.001) | 0% | 11% | 0.02 | 1.12 | 33.2 (.268) |
|  |  | Present (*k* = 13.95) | -0.02 (0.2) | -0.1 (.905) |  |  |  |  |  |
| Prophylactic ASM | Complete case  19 (5,622) | Intercept | 0.63 (0.2) | 3.4 (.003) | 0% | 0% | 0.00 | 1.00 | 16.8 (.470) |
|  |  | Present: 15 (4,596) | -0.11 (0.2) | -0.5 (.626) |  |  |  |  |  |
|  | Multiple imputation  *k* = 31 | Intercept | 0.61 (0.2) | 3.0 (.008) | 10% | 9% | 0.02 | 1.1 | 32.4 (.284) |
|  |  | Present (*k* = 23.75) | -0.06 (0.3) | -0.2 (.817) |  |  |  |  |  |
| Oedema Imaging | Complete case  23 (6,362) | Intercept | 0.98 (0.3) | 2.8 (.010) | 10% | 9% | 0.02 | 1.10 | 23.5 (.318) |
|  |  | MRI: 21 (6,130) | -0.41 (0.4) | -1.1 (.275) |  |  |  |  |  |
|  | Multiple imputation  *k* = 31 | Intercept | 0.86 (0.3) | 2.7 (.014) | 16% | 7% | 0.02 | 1.08 | 31.7 (.346) |
|  |  | MRI (*k* = 27.7) | -0.32 (0.3) | -0.9 (.356) |  |  |  |  |  |
| Oedema method  31 (7,776) | | Intercept | 0.5 (0.1) | 4 (<.001) | 0% | 10% | 0.02 | 1.11 | 32.7 (.290) |
|  |  | Binary: 17 (3,640) | 0.12 (0.2) | 0.7 (.467) |  |  |  |  |  |
| Bias  31 (7,776) | | Intercept | 0.49 (0.1) | 5 (<.001) | 25% | 6% | 0.01 | 1.07 | 31.3 (.354) |
|  |  | High/V.High: 12 (1,956) | 0.23 (0.2) | 1.3 (.199) |  |  |  |  |  |
| Variable *k* for multiple imputation is taken from complete case series and average number of ones from imputation. | | | | | | | | | |

### Online Resource 15: Multivariable meta-regression preoperative oedema and postoperative seizure

| Forced entry multivariable analysis | Multiple imputation  *k* = 31 | |
| --- | --- | --- |
| $R_{*}^{2}$ | 30% | |
| *F* (*p)*  *df*  *Coefficients* | - 1. (.458)   *df*1 = 7, *df*2 = 23  2:8 | |
| *I^2^* | 9% | |
| τ^2^ | 0.02 | |
| *H^2^* | 1.1 | |
| *QE*(*df*)p | 25.3 (23) .348 | |
| Study characteristic | *β (SE)* | *t* (*p*) |
| Intercept | 0.82 (0.6) | 1.5 (.171) |
| Late Postoperative seizure | 0.20 (0.4) | 0.4 (.668) |
| Preoperative seizure present | -0.18 (0.3) | -0.6 (.580) |
| Infratentorial tumours allowed | -0.15 (0.3) | -0.5 (.596) |
| Prophylactic ASM used | -0.13 (0.4) | -0.4 (.715) |
| MRI to measure oedema | -0.26 (0.5) | -0.6 (.582) |
| Binary oedema measurement | 0.07 (0.3) | 0.2 (.810) |
| High or v.High risk of bias | 0.26 (0.2) | 1.1 (.306) |
| *Only three studies had complete data so a complete case analysis was not performed* | | |

### Online Resource 16: Results of *p* curve analysis

| **Analysis** | **Test** | ***p* Binomial** | **Full curve** | | **Half curve** | | | **Evidential value** | | **Power estimate % (95% *CI*)** | **Cohen’s *d*** | ***OR^a^*** |
| --- | --- | --- | --- | --- | --- | --- | --- | --- | --- | --- | --- | --- |
|  |  |  | ***Z*** | ***p*** | ***Z*** | ***p*** | **Present** | | **Absent** |  |  |  |
| Preop oedema and preop seizure | Right skewness | .000 | -14.6 | <.001 | -14.4 | <.001 | Yes | | No | 99 (97-99) | 0.456 | 2.29 |
|  | Flatness | .992 | 10.3 | >.999 | 12.5 | >.999 |  |  |  |  |  |  |
| Preop oedema and preop seizure^b^ | Right skewness | .500 | -5.1 | <.001 | -6.5 | <.001 | Yes | | No | 98 (86-99) | 0.316 | 1.77 |
|  | Flatness | .636 | 3.5 | >.999 | 5.4 | >.999 |  |  |  |  |  |  |
| Preop oedema and all postop seizure | Right skewness | .500 | -2.1 | .019 | -2.1 | .019 | Yes | | No | 35 (7-72) | 0.121 | 1.25^c^ |
|  | Flatness | .239 | 0.1 | .534 | 2.8 | .998 |  |  |  |  |  |  |
| ^a^ *OR* converted from *d* using formula *OR* = antilog ($d\pi/\surd3)$  ^b^ Adjusted for headache and gender  ^c^ May be inaccurate, effect size search plot did not create ideal “V” | | | | | | | | | | | | |

### Online Resource 17: Variables controlled for in adjusted meta-analysis of preoperative seizure and preoperative oedema

| Variable included in multiple regression analysis | | Chaichana^46^ *n* = 626 | Chen^47^ *n* = 843 | Li & Wang^55^ *n* = 772 |
| --- | --- | --- | --- | --- |
| Core | Oedema | 1 | 1 | 1 |
|  | Male | 0 | 0 | 0 |
|  | Headache | -1 | -1 | -1 |
| Additional | Age |  | 1 | 1 |
|  | Performance status | -1 |  |  |
|  | Preoperative deficit |  | -1 | 0 |
|  | Convexity location | 0 |  |  |
|  | Skull base location |  | -1 | 0 |
|  | Suprasellar location | 0 |  |  |
|  | Midline location |  | 0 | 0 |
|  | Tumour size | 0 | 0 | 0 |
|  | WHO grade |  | 0 |  |
| 1 = significant positive association  0 = no association  -1 = significant negative association | | | | |

### Online Resource 18: Forest plot - re-analysis of preoperative oedema and preoperative seizure from Englot *et al.* ^43,46,53,56,100,105–108^

eh


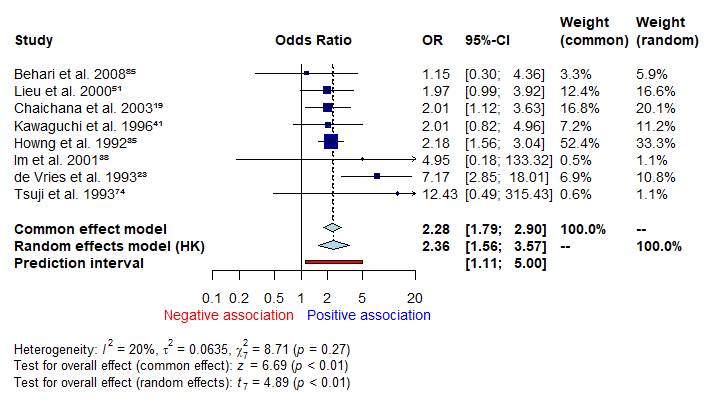


Tsuji

### Supplementary references

1. Balduzzi S, Rücker G, Schwarzer G. How to perform a meta-analysis with R: A practical tutorial. *Evidence-Based Mental Health*. 2019;22(4):153-160. doi:10.1136/ebmental-2019-300117

2. Harrer M, Cuijpers P, Furukawa T, Ebert DD. dmetar: Companion R Package For The Guide “Doing Meta-Analysis in R.” 2019. Accessed February 3, 2023. http://dmetar.protectlab.org/

3. Viechtbauer W. Conducting meta-analyses in R with the metafor package. *Journal of statistical software*. 2010;36(3):1-48.

4. Peterson B, Carl P. PerformanceAnalytics: Econometric Tools for Performance and Risk Analysis. Published online 2020.

5. Wickham H, Averick M, Bryan J, *et al*. Welcome to the Tidyverse Tidyverse package. 2019;4:1-6. doi:10.21105/joss.01686

6. van Buuren S, Groothuis-Oudshoorn K. mice: Multivariate imputation by chained equations in R. *Journal of Statistical Software*. 2011;45(3):1-67. doi:10.18637/jss.v045.i03

7. Hong Y, R Core Team. poibin: The Poisson Binomial Distribution. Published online 2020.

8. Wickham H. stringr: Simple, Consistent Wrappers for Common String Operations. Published online 2022.

9. Tierney N, Cook D. Expanding Tidy Data Principles to Facilitate Missing Data Exploration, Visualization and Assessment of Imputations. *Journal of Statistical Software*. 2023;105(7):1-31. doi:10.18637/jss.v105.i07

10. Abzalova DI, Sinkin M V., Yakovlev AA, Prirodov A V., Guekht AB. Risk Factors for the Development of De Novo Generalized Tonic-Clonic Epileptic Seizures in Patients with Supratentorial Meningiomas after Neurosurgical Treatment. *Neurosci Behav Physiol*. 2024;123(10):69-74. doi:10.1007/s11055-024-01605-x

11. Bogdanovic I, Ristic A, Ilic R, *et al*. Factors associated with preoperative and early and late postoperative seizures in patients with supratentorial meningiomas. *Epileptic Disorders*. 2023;25(2):244-254. doi:10.1002/epd2.20021

12. Spille DC, Hess K, Bormann E, *et al*. Risk of tumor recurrence in intracranial meningiomas: Comparative analyses of the predictive value of the postoperative tumor volume and the Simpson classification. *J Neurosurg*. 2021;134(6):1764-1771. doi:10.3171/2020.4.JNS20412

13. Cai Q, Wu Y, Wang S, *et al*. Preoperative antiepileptic drug prophylaxis for early postoperative seizures in supratentorial meningioma: a single-center experience. *J Neurooncol*. 2022;158(1):59-67. doi:10.1007/s11060-022-04009-4

14. Chaichana KL, Pendleton C, Zaidi H, *et al*. Seizure control for patients undergoing meningioma surgery. *World Neurosurg*. 2013;79(3-4):515-524. doi:10.1016/j.wneu.2012.02.051

15. McKevitt C, Marenco-Hillembrand L, Bamimore M, *et al*. Predictive factors for post operative seizures following meningioma resection in patients without preoperative seizures: a multicenter retrospective analysis. *Acta Neurochir (Wien)*. 2023;165(5):1333-1343. doi:10.1007/s00701-023-05571-0

16. Chen WC, Magill ST, Englot DJ, *et al*. Factors Associated with Pre- and Postoperative Seizures in 1033 Patients Undergoing Supratentorial Meningioma Resection. *Neurosurgery*. 2017;81(2):297-306. doi:10.1093/neuros/nyx001

17. de Vries J, Wakhloo AK. Cerebral oedema associated with WHO-I, WHO-II, and WHO-III-meningiomas: Correlation of clinical, computed tomographic, operative and histological findings. *Acta Neurochir (Wien)*. 1993;125(1-4):34-40. doi:10.1007/BF01401825

18. Ding D, Xu Z, McNeill IT, Yen CPP, Sheehan JP. Radiosurgery for parasagittal and parafalcine meningiomas: Clinical article. *J Neurosurg*. 2013;119(4):871-877. doi:10.3171/2013.6.JNS13110

19. Gadot R, Khan AB, Patel R, *et al*. Predictors of postoperative seizure outcome in supratentorial meningioma. *J Neurosurg*. 2022;137(2):515-524. doi:10.3171/2021.9.JNS211738

20. Gupte TP, Li C, Jin L, *et al*. Clinical and genomic factors associated with seizures in meningiomas. *J Neurosurg*. 2021;135(3):835-844. doi:10.3171/2020.7.JNS201042

21. Hamasaki T, Yamada K, Yano S, *et al*. Higher incidence of epilepsy in meningiomas located on the premotor cortex: A voxel-wise statistical analysis. *Acta Neurochir (Wien)*. 2012;154(12):2241-2249. doi:10.1007/s00701-012-1511-1

22. Hwang K, Joo JD, Kim YH, *et al*. Risk factors for preoperative and late postoperative seizures in primary supratentorial meningiomas. *Clin Neurol Neurosurg*. 2019;180(March):34-39. doi:10.1016/j.clineuro.2019.03.007

23. Islim AI, Ali A, Bagchi A, *et al*. Postoperative seizures in meningioma patients: improving patient selection for antiepileptic drug therapy. *J Neurooncol*. 2018;140(1):123-134. doi:10.1007/s11060-018-2941-2

24. Kemerdere R, Akgun MY, Alizada O, Toklu S, Tahmazoglu B, Tanriverdi T. Risk factors for preoperative and postoperative late seizure in supratentorial meningiomas. *Romanian Neurosurgery*. 2019;33(3):316-321. doi:10.33962/roneuro-2019-053

25. Lieu AS, Howng SL. Intracranial meningiomas and epilepsy: Incidence, prognosis and influencing factors. *Epilepsy Res*. 1999;38(1):45-52. doi:10.1016/S0920-1211(99)00066-2

26. Li X, Wang C, Lin Z, *et al*. Risk factors and control of seizures in 778 Chinese patients undergoing initial resection of supratentorial meningiomas. *Neurosurg Rev*. 2020;43(2):597-608. doi:10.1007/s10143-019-01085-5

27. Markovic M, Antunovic V, Milenkovic S, Zivkovic N. Prognostic value of peritumoral edema and angiogenesis in intracranial meningioma surgery. *Journal of BUON*. 2013;18(2):430-436.

28. Morsy M, El-Saadany W, Moussa W, Sultan A. Predictive factors for seizures accompanying intracranial meningiomas. *Asian J Neurosurg*. 2019;14(02):403-409. doi:10.4103/ajns.ajns_152_18

29. Teske N, Teske NC, Greve T, *et al*. Perifocal edema is a risk factor for preoperative seizures in patients with meningioma WHO grade 2 and 3. *Acta Neurochir (Wien)*. 2024;166(1). doi:10.1007/s00701-024-06057-3

30. Pauletto G, Nilo A, Pez S, *et al*. Meningioma-Related Epilepsy: A Happy Ending? *J Pers Med*. 2023;13(7). doi:10.3390/jpm13071124

31. Tsuji M, Shinomiya S, Inoue R, Sato K. Prospective study of postoperative seizure in intracranial meningioma. *Jpn J Psychiatry Neurol*. 1993;47(2):331-334.

32. Schneider M, Güresir Á, Borger V, *et al*. Preoperative tumor-associated epilepsy in patients with supratentorial meningioma: Factors influencing seizure outcome after meningioma surgery. *J Neurosurg*. 2020;133(6):1655-1661. doi:10.3171/2019.7.JNS19455

33. Wang YC, Chuang CC, Tu PH, *et al*. Seizures in surgically resected atypical and malignant meningiomas: Long-term outcome analysis. *Epilepsy Res*. 2018;140(September 2017):82-89. doi:10.1016/j.eplepsyres.2017.12.013

34. Singh G, Verma PK, Srivastava AK, *et al*. Factors predicting seizure outcome after surgical excision of meningioma: SOLID-C guideline for prophylactic AED. *Journal of Clinical Neuroscience*. 2023;117:143-150. doi:10.1016/j.jocn.2023.09.022

35. Wirsching HG, Morel C, Gmür C, *et al*. Predicting outcome of epilepsy after meningioma resection. *Neuro Oncol*. 2016;18(7):1002-1010. doi:10.1093/neuonc/nov303

36. Stevens JM, Ruiz JS, Kendall BE. Observations on peritumoural oedema in meningioma - Part II: Mechanisms of oedema production. *Neuroradiology*. 1983;25(3):125-131. doi:10.1007/BF00455731

37. Yang M, Cheng YR, Zhou MY, *et al*. Prophylactic AEDs Treatment for Patients With Supratentorial Meningioma Does Not Reduce the Rate of Perioperative Seizures: A Retrospective Single-Center Cohort Study. *Front Oncol*. 2020;10(December):1-8. doi:10.3389/fonc.2020.568369

38. Zhang P, Li Y, Zhang J, *et al*. Risk factors analysis and a nomogram model establishment for late postoperative seizures in patients with meningioma. *Journal of Clinical Neuroscience*. 2020;80:310-317. doi:10.1016/j.jocn.2020.06.005

39. Zhang B, Wang D, Guo Y, YU J. Clinical multifactorial analysis of early postoperative seizures in elderly patients following meningioma resection. *Mol Clin Oncol*. 2015;3(3):501-505. doi:10.3892/mco.2015.493

40. Markovic M, Antunovic V, Milenkovic S, Zivkovic N. Prognostic value of peritumoral edema and angiogenesis in intracranial meningioma surgery. *Journal of BUON*. 2013;18(2):430-436.

41. Stevens JM, Ruiz JS, Kendall BE. Observations on peritumoural oedema in meningioma - Part II: Mechanisms of oedema production. *Neuroradiology*. 1983;25(3):125-131. doi:10.1007/BF00455731

42. Seyedi JF, Pedersen CB, Poulsen FR. Risk of seizures before and after neurosurgical treatment of intracranial meningiomas. *Clin Neurol Neurosurg*. 2018;165:60-66. doi:10.1016/j.clineuro.2018.01.002

43. Howng SL, Kwan AL. Intracranial meningioma. *Kaohsiung J Med Sci*. 1992;8(6):312-319.

44. Baumgarten P, Sarlak M, Monden D, *et al*. Early and late postoperative seizures in meningioma patients and prediction by a recent scoring system. *Cancers (Basel)*. 2021;13(3):1-13. doi:10.3390/cancers13030450

45. Bogdanovic I, Ristic A, Ilic R, *et al*. Factors associated with preoperative and early and late postoperative seizures in patients with supratentorial meningiomas. *Epileptic Disorders*. 2023;25(2):244-254. doi:10.1002/epd2.20021

46. Chaichana KL, Pendleton C, Zaidi H, *et al*. Seizure control for patients undergoing meningioma surgery. *World Neurosurg*. 2013;79(3-4):515-524. doi:10.1016/j.wneu.2012.02.051

47. Chen WC, Magill ST, Englot DJ, *et al*. Factors Associated with Pre- and Postoperative Seizures in 1033 Patients Undergoing Supratentorial Meningioma Resection. *Neurosurgery*. 2017;81(2):297-306. doi:10.1093/neuros/nyx001

48. Gupte TP, Li C, Jin L, *et al*. Clinical and genomic factors associated with seizures in meningiomas. *J Neurosurg*. 2021;135(3):835-844. doi:10.3171/2020.7.JNS201042

49. Hamasaki T, Yamada K, Yano S, *et al*. Higher incidence of epilepsy in meningiomas located on the premotor cortex: A voxel-wise statistical analysis. *Acta Neurochir (Wien)*. 2012;154(12):2241-2249. doi:10.1007/s00701-012-1511-1

50. Hinrichs FL, Brokinkel C, Adeli A, *et al*. Risk factors for preoperative seizures in intracranial meningiomas. *J Neurosurg Sci*. 2023;67(1):66-72. doi:10.23736/S0390-5616.20.05068-7

51. Hwang K, Joo JD, Kim YH, *et al*. Risk factors for preoperative and late postoperative seizures in primary supratentorial meningiomas. *Clin Neurol Neurosurg*. 2019;180(March):34-39. doi:10.1016/j.clineuro.2019.03.007

52. Islim AI, Ali A, Bagchi A, *et al*. Postoperative seizures in meningioma patients: improving patient selection for antiepileptic drug therapy. *J Neurooncol*. 2018;140(1):123-134. doi:10.1007/s11060-018-2941-2

53. Kawaguchi T, Kameyama S, Tanaka R. Peritumoral edema and seizures in patients with cerebral convexity and parasagittal meningiomas. *Neurol Med Chir (Tokyo)*. 1995;35(568):574. doi:10.2176/nmc.36.568

54. Kemerdere R, Akgun MY, Alizada O, Toklu S, Tahmazoglu B, Tanriverdi T. Risk factors for preoperative and postoperative late seizure in supratentorial meningiomas. *Romanian Neurosurgery*. 2019;33(3):316-321. doi:10.33962/roneuro-2019-053

55. Li X, Wang C, Lin Z, *et al*. Risk factors and control of seizures in 778 Chinese patients undergoing initial resection of supratentorial meningiomas. *Neurosurg Rev*. 2020;43(2):597-608. doi:10.1007/s10143-019-01085-5

56. Lieu AS, Howng SL. Intracranial meningiomas and epilepsy: Incidence, prognosis and influencing factors. *Epilepsy Res*. 1999;38(1):45-52. doi:10.1016/S0920-1211(99)00066-2

57. Morsy M, El-Saadany W, Moussa W, Sultan A. Predictive factors for seizures accompanying intracranial meningiomas. *Asian J Neurosurg*. 2019;14(02):403-409. doi:10.4103/ajns.ajns_152_18

58. Nassar A, Smolanka V, Taras S, *et al*. Risk factors for preoperative seizures in meningiomas - base versus non-bases of supratentorial . Single centre retrospective study in a series of 244 cases. *Romanian Neurosurgery*. 2022;36(2):237-246. doi:10.33962/roneuro-2022-042

59. Pauletto G, Nilo A, Pez S, *et al*. Meningioma-Related Epilepsy: A Happy Ending? *J Pers Med*. 2023;13(7). doi:10.3390/jpm13071124

60. Teske N, Teske NC, Greve T, *et al*. Perifocal edema is a risk factor for preoperative seizures in patients with meningioma WHO grade 2 and 3. *Acta Neurochir (Wien)*. 2024;166(1). doi:10.1007/s00701-024-06057-3

61. Wach J, Güresir Á, Vatter H, *et al*. Low-Dose Acetylsalicylic Acid Treatment in Non-Skull-Base Meningiomas: Impact on Tumor Proliferation and Seizure Burden. *Cancers (Basel)*. 2022;14(17):1-15. doi:10.3390/cancers14174285

62. Wang YC, Chuang CC, Tu PH, *et al*. Seizures in surgically resected atypical and malignant meningiomas: Long-term outcome analysis. *Epilepsy Res*. 2018;140(September 2017):82-89. doi:10.1016/j.eplepsyres.2017.12.013

63. Wirsching HG, Morel C, Gmür C, *et al*. Predicting outcome of epilepsy after meningioma resection. *Neuro Oncol*. 2016;18(7):1002-1010. doi:10.1093/neuonc/nov303

64. Abzalova DI, Sinkin M V., Yakovlev AA, Prirodov A V., Guekht AB. Risk Factors for the Development of De Novo Generalized Tonic-Clonic Epileptic Seizures in Patients with Supratentorial Meningiomas after Neurosurgical Treatment. *Neurosci Behav Physiol*. 2024;123(10):69-74. doi:10.1007/s11055-024-01605-x

65. Cai Q, Wu Y, Wang S, *et al*. Preoperative antiepileptic drug prophylaxis for early postoperative seizures in supratentorial meningioma: a single-center experience. *J Neurooncol*. 2022;158(1):59-67. doi:10.1007/s11060-022-04009-4

66. Xu J, Yu Y, Li Q, *et al*. Radiomic features as a risk factor for early postoperative seizure in patients with meningioma. *Seizure*. 2021;93(April):120-126. doi:10.1016/j.seizure.2021.10.012

67. Zhang P, Li Y, Zhang J, *et al*. Risk factors analysis and a nomogram model establishment for late postoperative seizures in patients with meningioma. *Journal of Clinical Neuroscience*. 2020;80:310-317. doi:10.1016/j.jocn.2020.06.005

68. Yang M, Cheng YR, Zhou MY, *et al*. Prophylactic AEDs Treatment for Patients With Supratentorial Meningioma Does Not Reduce the Rate of Perioperative Seizures: A Retrospective Single-Center Cohort Study. *Front Oncol*. 2020;10(December):1-8. doi:10.3389/fonc.2020.568369

69. Ahmeti H, Caliebe A, Röcken C, Jansen O, Mehdorn MH, Synowitz M. Impact of peritumoral brain edema on pre- and postoperative clinical conditions and on long-term outcomes in patients with intracranial meningiomas. *Eur J Med Res*. 2023;28(1):1-15. doi:10.1186/s40001-022-00962-y

70. Zhang B, Wang D, Guo Y, YU J. Clinical multifactorial analysis of early postoperative seizures in elderly patients following meningioma resection. *Mol Clin Oncol*. 2015;3(3):501-505. doi:10.3892/mco.2015.493

71. Zhang J, Cao Y, Zhang G, *et al*. Nomogram based on MRI can preoperatively predict brain invasion in meningioma. *Neurosurgical Review*. 2022;45(6):3729-3737. doi:10.1007/s10143-022-01872-7

72. Ahmed AK, Wilhelmy B, Oliver J, *et al*. Variability in the Arterial Supply of Intracranial Meningiomas: An Anatomic Study. *Neurosurgery*. 2023;93(6):1346-1352. doi:10.1227/neu.0000000000002608

73. Goertz L, Hamisch C, Erdner N, *et al*. Independent risk factors for pre- and postoperative seizures in meningioma patients identified by logistic regression analysis. *Neuro Oncol*. 2018;20(supplement 3):ii319. doi:10.1093/neuonc/noy139.395

74. Lobato RD, Alday R, Gómez PA, *et al*. Brain Oedema in Patients with Intracranial Meningioma: Correlation between Clinical, Radiological, and Histological Factors and the Presence and Intensity of Oedema. *Acta Neurochir (Wien)*. 1996;138(5):485-494. doi:10.1007/BF01411166

75. Loewenstern J, Aggarwal A, Pain M, *et al*. Peritumoral edema relative to meningioma size predicts functional outcomes after resection in older patients. *Operative Neurosurgery*. 2019;16(3):281-291. doi:10.1093/ons/opy107

76. Salpietro F, Alafaci C, Lucerna S, Matalone D, Morabito G. Peritumoral edema and seizures in patients with cerebral convexity and parasagittal meningiomas. *Ital J Neurol Sci*. 1997;18(4):255.

77. Simis A, Pires de Aguiar PH, Leite CC, Santana PA, Rosemberg S, Teixeira MJ. Peritumoral brain edema in benign meningiomas: correlation with clinical, radiologic, and surgical factors and possible role on recurrence. *Surg Neurol*. 2008;70(5):471-477. doi:10.1016/j.surneu.2008.03.006

78. Ersoy TF, Ridwan S, Grote A, Coras R, Simon M. Early postoperative seizures (EPS) in patients undergoing brain tumour surgery. *Sci Rep*. 2020;10(1):1-10. doi:10.1038/s41598-020-70754-z

79. Lazzarin S, Impellizzeri M, Barzaghi L, *et al*. Short-term outcomes and predictors of acute postoperative seizures in patients undergoing supratentorial craniotomy. *Neurological Sciences*. 2022;43(Supplement 1):s283.

80. Asemota AO, Huang LH, Boling W. Thirty-Day Readmissions and Seizure Risk after Surgical Resection of Intracranial Meningiomas: Analysis of a National Database. *J Neurol Surg B Skull Base*. 2022;83(Supplement 1):S1-S270. doi:10.1055/s-0042-1743749

81. Goertz L, Bernards N, Muders H, Hamisch C, Goldbrunner R, Krischek B. Incidence and Clinical Presentation of Pre- and Postoperative Seizures in Patients With Posterior Fossa Meningiomas. *Cureus*. 2024;16(1):1-8. doi:10.7759/cureus.52474

82. Hess K, Spille DC, Adeli A, *et al*. Brain invasion and the risk of seizures in patients with meningioma. *J Neurosurg*. 2019;130(3):789-796. doi:10.3171/2017.11.JNS172265

83. Le VT, Nguyen AM, Pham TA, Nguyen PL. Tumor-related epilepsy and post-surgical outcomes: tertiary hospital experience in Vietnam. *Sci Rep*. 2023;13(1):1-9. doi:10.1038/s41598-023-38049-1

84. Xue H, Sveinsson O, Bartek J, *et al*. Long-term control and predictors of seizures in intracranial meningioma surgery: a population-based study. *Acta Neurochir (Wien)*. 2018;160(3):589-596. doi:10.1007/s00701-017-3434-3

85. Kirn JH, Park K, Nom DH, Shin HJ. Peritumoral edema in meningioma: Correlation to clinical presentation and surgical outcome. *Skull Base Surg*. 1998;8(Supplement 1):21.

86. Skardelly M, Rother C, Noell S, *et al*. Risk Factors of Preoperative and Early Postoperative Seizures in Patients with Meningioma: A Retrospective Single-Center Cohort Study. *World Neurosurg*. 2017;97:538-546. doi:10.1016/j.wneu.2016.10.062

87. Conti A, Pontoriero A, Siddi F, *et al*. Post-Treatment Edema after Meningioma Radiosurgery is a Predictable Complication. *Cureus*. 2016;8(5):1-11. doi:10.7759/cureus.605

88. Jung IH, Chang KW, Park SH, *et al*. Pseudoprogression and peritumoral edema due to intratumoral necrosis after Gamma knife radiosurgery for meningioma. *Sci Rep*. 2022;12(1):1-10. doi:10.1038/s41598-022-17813-9

89. Patil CG, Hoang S, Borchers DJ, *et al*. Predictors of peritumoral edema after stereotactic radiosurgery of supratentorial meningiomas. *Neurosurgery*. 2008;63(3):435-440. doi:10.1227/01.NEU.0000325257.58684.92

90. Zachenhofer I, Wolfsberger S, Aichholzer M, *et al*. Gamma-knife radiosurgery for cranial base meningiomas: Experience of tumor control, clinical course, and morbidity in a follow-up of more than 8 years. *Neurosurgery*. 2006;58(1):28-36. doi:10.1227/01.NEU.0000190654.82265.A3

91. Brokinkel B, Hinrichs FL, Schipmann S, *et al*. Predicting postoperative seizure development in meningiomas – Analyses of clinical, histological and radiological risk factors. *Clin Neurol Neurosurg*. 2021;200(September 2020). doi:10.1016/j.clineuro.2020.106315

92. Frati A, Armocida D, Arcidiacono UA, *et al*. Peritumoral Brain Edema in Relation to Tumor Size Is a Variable That Influences the Risk of Recurrence in Intracranial Meningiomas. *Tomography*. 2022;8(4):1987-1996. doi:10.3390/tomography8040166

93. Gadot R, Khan AB, Patel R, *et al*. Predictors of postoperative seizure outcome in supratentorial meningioma. *J Neurosurg*. 2022;137(2):515-524. doi:10.3171/2021.9.JNS211738

94. Güngör A, Danyeli AE, Akbaş A, *et al*. Ventricular Meningiomas: Surgical Strategies and a New Finding That Suggest an Origin From the Choroid Plexus Epithelium. *World Neurosurg*. 2019;129:e177-e190. doi:10.1016/j.wneu.2019.05.092

95. McKevitt C, Marenco-Hillembrand L, Bamimore M, *et al*. Predictive factors for post operative seizures following meningioma resection in patients without preoperative seizures: a multicenter retrospective analysis. *Acta Neurochir (Wien)*. 2023;165(5):1333-1343. doi:10.1007/s00701-023-05571-0

96. Nassar A, Smolanka V, Smolanka A, Chaulagain D, Devinyak O. Sphenoid wing meningiomas: peritumoral brain edema as a prognostic factor in surgical outcome. *Neurosurg Rev*. 2022;45(4):2951-2959. doi:10.1007/s10143-022-01816-1

97. Rajab YS, Aji AO, Abdin AJ, Alkharrat L. *A Retrospective Cohort Study of Risk of Seizures in Pre and Postoperative Treatment of Supratentorial Meningioma*.; 2022. doi:10.21203/rs.3.rs-2219350/v1

98. Schneider M, Güresir Á, Borger V, *et al*. Preoperative tumor-associated epilepsy in patients with supratentorial meningioma: Factors influencing seizure outcome after meningioma surgery. *J Neurosurg*. 2020;133(6):1655-1661. doi:10.3171/2019.7.JNS19455

99. Singh G, Verma PK, Srivastava AK, *et al*. Factors predicting seizure outcome after surgical excision of meningioma: SOLID-C guideline for prophylactic AED. *Journal of Clinical Neuroscience*. 2023;117:143-150. doi:10.1016/j.jocn.2023.09.022

100. Tsuji M, Shinomiya S, Inoue R, Sato K. Prospective study of postoperative seizure in intracranial meningioma. *Jpn J Psychiatry Neurol*. 1993;47(2):331-334.

101. Kollová A, Liščák R, Novotný J, Vladyka V, Šimonová G, Janoušková L. Gamma Knife surgery for benign meningioma. *J Neurosurg*. 2007;107(2):325-336. doi:10.3171/JNS-07/08/0325

102. Ding D, Xu Z, McNeill IT, Yen CPP, Sheehan JP. Radiosurgery for parasagittal and parafalcine meningiomas: Clinical article. *J Neurosurg*. 2013;119(4):871-877. doi:10.3171/2013.6.JNS13110

103. Hwang K, Kim DG, Paek SH, *et al*. Seizures After Stereotactic Radiosurgery for Benign Supratentorial Meningiomas: An Uncontrollable Type of Seizure? *World Neurosurg*. 2019;123:e549-e556. doi:10.1016/j.wneu.2018.11.211

104. Kuhn EN, Taksler GB, Dayton O, *et al*. Is there a tumor volume threshold for postradiosurgical symptoms? a single-institution analysis. *Neurosurgery*. 2014;75(5):536-544. doi:10.1227/NEU.0000000000000519

105. Behari S, Giri PJ, Shukla D, Jain VK, Deepu B. Surgical strategies for giant medial sphenoid wing meningiomas : a new scoring system for predicting extent of resection. *Acta Neurochir (Wien)*. 2008;150:865-877. doi:10.1007/s00701-008-0006-6

106. Im SH, Wang KC, Kim SK, *et al*. Childhood meningioma: Unusual location, atypical radiological findings, and favorable treatment outcome. *Child’s Nervous System*. 2001;17(11):656-662. doi:10.1007/s003810100507

107. de Vries J, Wakhloo AK. Cerebral oedema associated with WHO-I, WHO-II, and WHO-III-meningiomas: Correlation of clinical, computed tomographic, operative and histological findings. *Acta Neurochir (Wien)*. 1993;125(1-4):34-40. doi:10.1007/BF01401825

108. Englot DJ, Magill ST, Han SJ, Chang EF, Berger MS, McDermott MW. Seizures in supratentorial meningioma: A systematic review and meta-analysis. *J Neurosurg*. 2016;124(6):1552-1561. doi:10.3171/2015.4.JNS142742.
